# *SERPING1* Variants and C1-INH Biological Function: A Close Relationship With C1-INH-HAE

**DOI:** 10.3389/falgy.2022.835503

**Published:** 2022-03-31

**Authors:** Christian Drouet, Alberto López-Lera, Arije Ghannam, Margarita López-Trascasa, Sven Cichon, Denise Ponard, Faidra Parsopoulou, Hana Grombirikova, Tomáš Freiberger, Matija Rijavec, Camila L. Veronez, João Bosco Pesquero, Anastasios E. Germenis

**Affiliations:** ^1^Department of Infection, Immunity and Inflammation, Institut Cochin, INSERM UMR1016, Université de Paris, Paris, France; ^2^Univ. Grenoble-Alpes & Centre Hospitalier Universitaire de Grenoble, Grenoble, France; ^3^Hospital La Paz Institute for Health Research (IdiPAZ), CIBERER U-754, Madrid, Spain; ^4^KininX SAS, Grenoble, France; ^5^Hospital La Paz Institute for Health Research (IdiPAZ), Universidad Autónoma de Madrid, Madrid, Spain; ^6^Human Genomics Research Group, Department of Biomedicine, University of Basel, Basel, Switzerland; ^7^Institute of Medical Genetics and Pathology, University Hospital Basel, Basel, Switzerland; ^8^Centre Hospitalier Universitaire de Grenoble, Grenoble, France; ^9^CeMIA SA, Larissa, Greece; ^10^Molecular Genetics Laboratory, Centre for Cardiovascular Surgery and Transplantation Brno and Medical Faculty, Masaryk University, Brno, Czechia; ^11^University Clinic of Respiratory and Allergic Diseases Golnik, Golnik, Slovenia; ^12^Department of Biophysics, Centre for Research and Genetic Diagnosis of Genetic Diseases, Federal University of São Paolo, São Paolo, Brazil; ^13^Department of Immunology & Histocompatibility, School of Health Sciences, Faculty of Medicine, University of Thessaly, Larissa, Greece

**Keywords:** C1-INH-HAE, C1 Inhibitor, *SERPING1* gene, genetic variation, serpin function, serpinopathy, angioedema, hereditary–diagnosis

## Abstract

Hereditary angioedema with C1 Inhibitor deficiency (C1-INH-HAE) is caused by a constellation of variants of the *SERPING1* gene (*n* = 809; 1,494 pedigrees), accounting for 86.8% of HAE families, showing a pronounced mutagenic liability of *SERPING1* and pertaining to 5.6% *de novo* variants. C1-INH is the major control serpin of the kallikrein–kinin system (KKS). In addition, C1-INH controls complement C1 and plasminogen activation, both systems contributing to inflammation. Recognizing the failed control of C1s protease or KKS provides the diagnosis of C1-INH-HAE. *SERPING1* variants usually behave in an autosomal-dominant character with an incomplete penetrance and a low prevalence. A great majority of variants (809/893; 90.5%) that were introduced into online database have been considered as pathogenic/likely pathogenic. Haploinsufficiency is a common feature in C1-INH-HAE where a dominant-negative variant product impacts the wild-type allele and renders it inactive. Small (36.2%) and large (8.3%) deletions/duplications are common, with exon 4 as the most affected one. Point substitutions with missense variants (32.2%) are of interest for the serpin structure–function relationship. Canonical splice sites can be affected by variants within introns and exons also (14.3%). For noncanonical sequences, exon skipping has been confirmed by splicing analyses of patients' blood-derived RNAs (*n* = 25). Exonic variants (*n* = 6) can affect exon splicing. Rare deep-intron variants (*n* = 6), putatively acting as pseudo-exon activating mutations, have been characterized as pathogenic. Some variants have been characterized as benign/likely benign/of uncertain significance (*n* = 74). This category includes some homozygous (*n* = 10) or compound heterozygous variants (*n* = 11). They are presenting with minor allele frequency (MAF) below 0.00002 (i.e., lower than C1-INH-HAE frequency), and may be quantitatively unable to cause haploinsufficiency. Rare benign variants could contribute as disease modifiers. Gonadal mosaicism in C1-INH-HAE is rare and must be distinguished from a *de novo* variant. Situations with paternal or maternal disomy have been recorded (*n* = 3). Genotypes must be interpreted with biological investigation fitting with C1-INH expression and typing. Any *SERPING1* variant reminiscent of the dysfunctional phenotype of serpin with multimerization or latency should be identified as serpinopathy.

## Introduction

**Figure 1:**
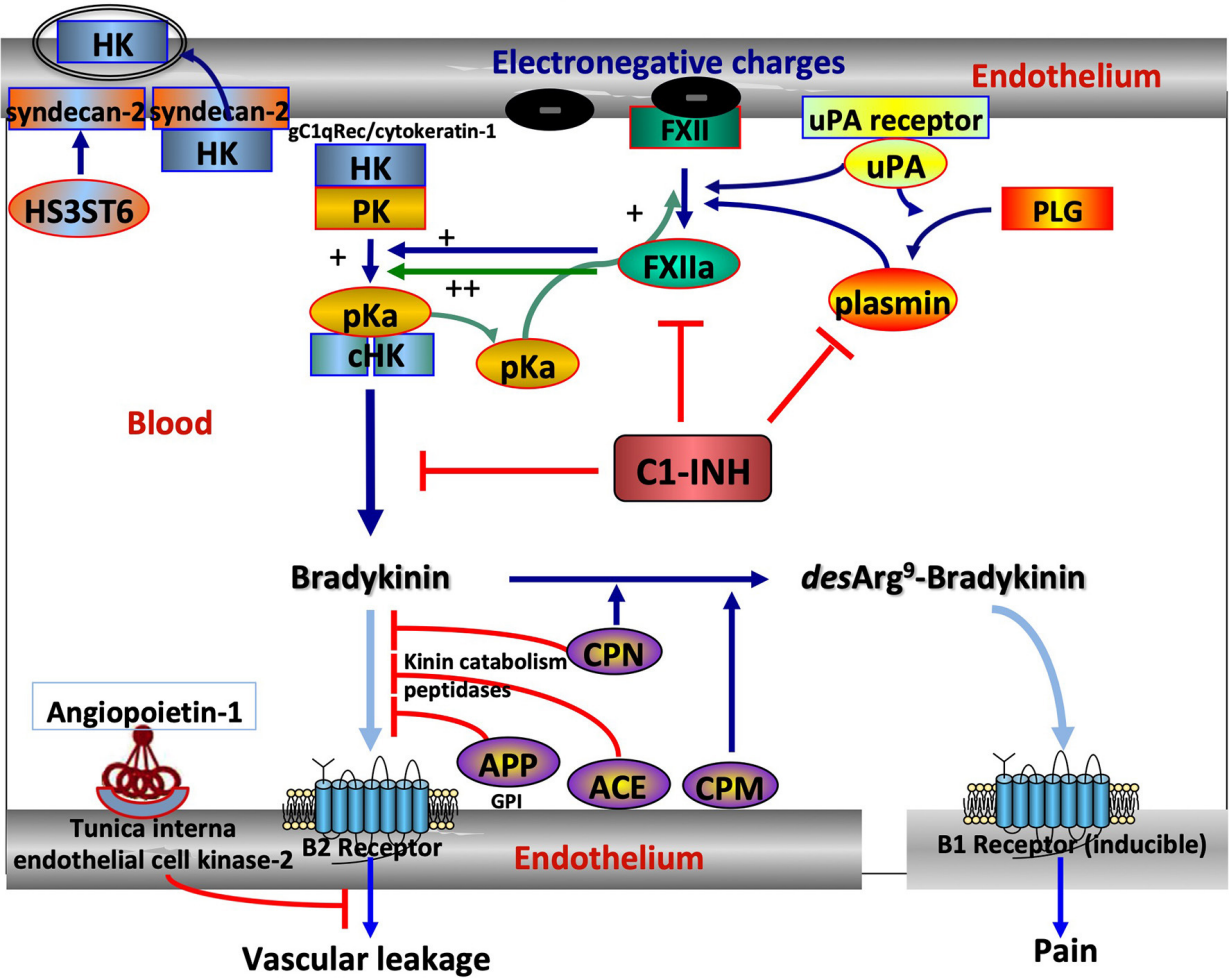
The central position of C1 Inhibitor (C1-INH) within the kallikrein–kinin system (KKS) and its companions. KKS activation is triggered when FXII becomes activated into FXIIa on binding to an activator onto an electronegative membrane. KKS activation results in HK cHK and bradykinin (BK). The serpin C1-INH controls both activation and activity of KKS, with zymogen to enzyme conversion of FXII and pK. C1-INH also controls the reciprocal activation of FXII and pK by plasmin in an interconnected amplification (5). ANGPT1 is a secreted protein ligand for tunica interna endothelium kinase-2, a receptor expressed in growing vascular endothelial cells. ANGPT1 targets key mechanisms contributing to the maintenance of endothelium function by inhibiting the effects of permeability enhancing agents, including BK, protecting from extensive permeability. H3ST6 is involved in HK docking on the endothelial cell surface, preventing HK binding to gC1q receptor and cytokeratin-1 and engaging in kinin forming. Aminopeptidase P (APP) and Angiotensin-I converting enzyme (ACE) are the membrane peptidases that inactivate BK, whereas carboxypeptidases M and N transform a B2 ligand into a B1 ligand. What this scheme means for understanding hereditary angioedema (HAE). *SERPING1* variants display a markedly reduced control function of C1-INH toward KKS and plasmin. *F12* and *PLG* variants are shown to increase FXII and plasminogen (PLG) activation, respectively, and *KNG1* variants more susceptible to HK cleavage with BK production. *ANGPT1* variants showed reduced capacity to bind its natural receptor, with less control of BK-dependent vascular leakage. Because of incomplete heparan-sulfate modification of syndecan-2 by H3ST6, *H3ST6* variants were less able to take up HK *via* endocytosis into the endothelial cell and more HKs entering into the KKS process. Dark blue arrows indicate activation, green arrows the amplification loop, light blue arrows the ligand–receptor interactions, and red lines indicate an inactivation of BK function. FXII, Factor XII; pK, prekallikrein; pKa, plasma kallikrein; HK, High-molecular-weight kininogen; cHK, cleaved HK; ANGPT1, angiopoietin-1; H3ST6, Heparan-sulfate-glucosamine 3-*O*-sulfotransferase 6; uPA, urokinase-type plasminogen activator.

Knowledge on angioedema has been first aimed at unraveling the pathophysiology of hereditary angioedema (HAE) with C1 Inhibitor (C1-INH) deficiency, namely, C1-INH-HAE (OMIM #106100; ORPHANET #91,378), a rare genetic disorder that is inherited as an autosomal-dominant trait in most cases. This prototypical condition has been shown to be bradykinin (BK) mediated by a clinical response to a specific BK receptor antagonist (1). Its prevalence is low, from 1/50,000 to 1/100,000, without known ethnic differences (2). HAE pathogenesis has been progressively deciphered from patient observations and basic investigations, showing that HAE results from variants in the *SERPING1* gene in association with the dysfunction of C1-INH (3). When the function of C1-INH has failed, circulating kallikrein–kinin system (KKS) is insufficiently controlled, with subsequent prekallikrein to plasma kallikrein conversion and the production of BK, a vasoactive peptide that causes increased vascular permeability with an activation of the B2 BK receptor. Together with KKS, complement and fibrinolysis are under C1-INH control, all systems sharing many inflammatory features are mediated by the vascular system (4). Figure 1 shows the central position of C1-INH in the control of kinin forming systems and the additional companions that could be affected by pathogenic variants.

Complement activation is not documented as it is directly involved in HAE pathophysiology. However, plasma kallikrein has been recognized as a pro-convertase, with anaphylatoxin production (6). In addition to anaphylatoxin generation, complement proteases promote plasminogen activation (7), with plasmin production that in turn triggers KKS. This latter proteolytic system directly cleaves circulating high molecular weight kininogen, subsequently generating BK production. Reversely, plasmin has been demonstrated to activate the key complement proteins C3 and C5 (8). These observations provide arguments for an interplay between complement, KKS, and fibrinolysis (4, 9), sustaining inflammation with a pivotal control by C1-INH. Furthermore, medications targeting the KKS or a B2 BK receptor have been developed and opened a way to understand HAE pathogenesis (10).

An important issue in C1-INH-HAE is the relationship between the systemic plasmatic changes in the KKS activation process and the local effect of BK accumulation in angioedema attacks (11). A failed C1-INH function has been considered as a causative participant. However, other contributors might be critical for the disease severity risk [e.g., kinin catabolism (12) and neutrophil inflammatory mediators (13, 14)] or for an angioedema phenotype of upper airway [e.g., EBV infection (15)]. The contribution of peripheral blood mononuclear cells to the HAE clinical phenotype has been questioned (16). In practice, novel genetic strategies are emerging, resulting in the characterization of a combination of common variants in *SERPING1* and in other genes involved in kinin pathway and metabolism (17).

Many observations of families carrying *SERPING1* variants and associated C1-INH data have been collected. This study aims to figure out the state-of-the art of *SERPING1* genetics, with an advantage for C1-INH-HAE diagnostic and its relationship with C1-INH expression and function. Recognizing C1-INH features and *SERPING1* genetics together is a prerequisite for the curation of variant pathogenicity.

## C1-INH Deficiency

The diagnosis of HAE with C1-INH deficiency (C1-INH-HAE) is established on a decreased C1-INH function. Rosen et al. distinguished a type-1 HAE (HAE-1), where C1-INH-HAE results from the failure to synthesize the protein, from a type-2 HAE (HAE-2) where an abnormal, dysfunctional protein is synthesized (18). HAE-2 is commonly identified from the data presenting with normal, or elevated, antigenic C1-INH in serum (19). However, many dysfunctional missense variants with a low antigenic C1-INH have been shown to be expressed together with the normal allele (20); they are characterized as HAE-2. Sharing same clinical presentation, diagnostic and pathophysiology, both types are also sharing the same recommendations for treatment options (10). This suggests that a distinction between HAE-1 and HAE-2 should not be relevant for physician tasks, but could be valuable for a structure–function relationship in the identification of pedigrees.

Substantial misdiagnoses and the delayed diagnoses of C1-INH-HAE are common. A significant obstacle for diagnosis is its low prevalence, high phenotypic variability, and incomplete penetrance, with the failed suspicion of angioedema symptoms, in particular abdominal attacks (21). This emphasizes the need to identify patients with HAE and HAE families to improve disease management and patient outcome (22).

**Figure 2:**
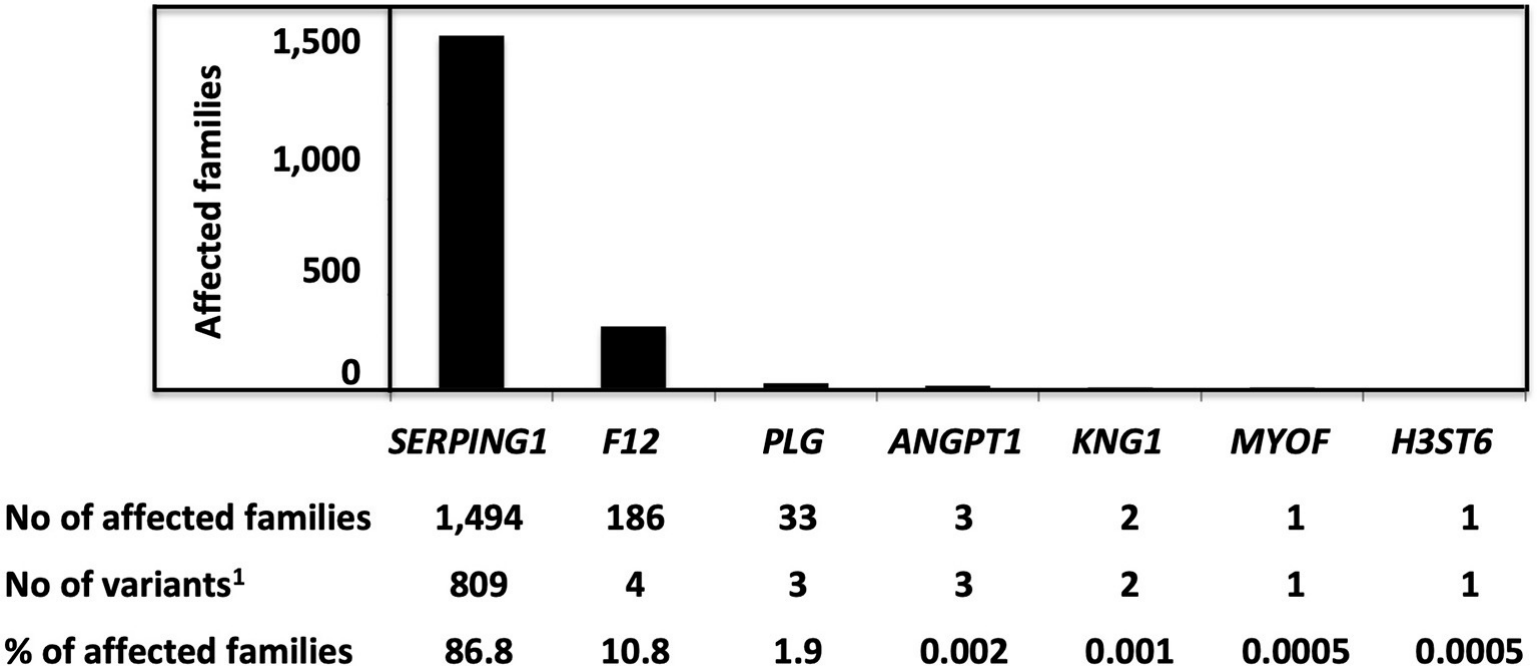
The distribution of variants responsible for HAE and characterized as pathogenic/likely pathogenic, with a number of affected families. Pathogenic/likely pathogenic variants have been characterized in agreement with the American College of Medical Genetics (ACMG) criteria or as declared by authors in the observations. ^1^Variants were characterized as pathogenic/likely pathogenic.

Hereditary angioedema due to C1 Inhibitor deficiency shares a common kinin dependency with other HAE situations: F12-HAE, PLG-HAE, KNG1-HAE, ANGPT1-HAE, and HS3ST6-HAE, but not with MYOF-HAE, U-HAE (Figure 1). The distribution of HAE conditions is shown in Figure 2. With 1,494 affected families recorded so far, C1-INH-HAE represents a predominant HAE condition (86.8%). Variants of these HAE-associated genes have been characterized with autosomal-dominant transmission and incomplete penetrance. Some rare *SERPING1* variants have been found in affected families with a recessive transmission (Section Recessive Variants), and *de novo* variants have been identified in 5.6% of the probands (20). These C1-INH-HAE features prompt biologists to perform the most accurate assays of the function of C1-INH for analytical HAE diagnostics.

### The Function of C1-INH and Its Analysis in Plasma

C1 Inhibitor is a multifunctional serine protease inhibitor (serpin) that controls various serine proteases involved in multiple plasmatic cascades [e.g., KKS, thereby limiting the production of the vasoactive peptide BK (23)]. C1-INH is a single-chain, highly glycosylated circulating protein of M*r* 105 kDa (nonreducing conditions) and 478 aminoacid residues (24).

**Figure 3:**
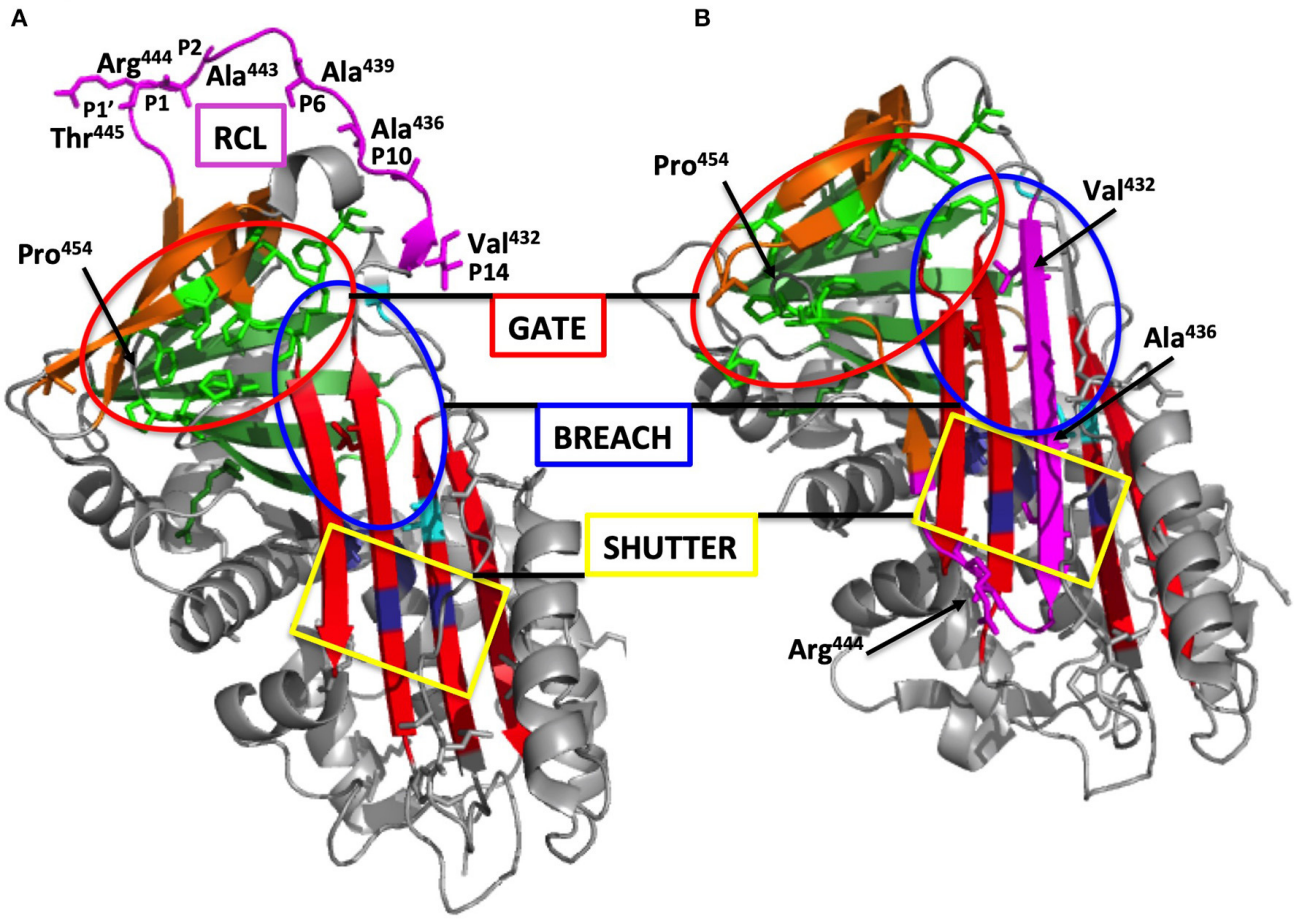
Overall structure of C1-INH. **(A)** Native C1-INH serpin domain, with positions of strategic residues shown with side chains in sticks. Five regions for the serpin function are presented on the 3D-model of C1Inh (PDB ID 5DU3) presented using Pymol. The model starts at Phe^100^ and lacks a great part of the N-terminal domain (residues 1-112). Strategic functional regions are indicated with (i) the *reactive site loop* (RCL), colored purple, including Arg^444^ P1 and the *hinge region* (P15-P9), essential for protease recognition, RCL mobility and conformational transformation for its insertion as strand 4A (s4A) (i.e., S to R transition, after protease inhibition); (ii) the *central* β*-sheet A*, colored red, with the *breach* region, indicated by a blue ellipse, located at its top and point of initial insertion of RCL, and the *shutter* domain, with a yellow rectangle, close to the center of β-sheet A, that, with *breach*, assists sheet opening and accepts the insertion of conserved *proximal hinge* s4A between s3A and s5A; (iii) the *gate*, highlighted with green sticks and targeted with a red ellipse, including s3C and s4C of β-sheet C. β-sheets B and C are colored green and brown, respectively. **(B)** Latent form of C1-INH. The same regions involved in the serpin function but in a nonfunctional conformation where RCL is kept inside the central β-sheet A structure with additional s4A colored purple and remaining inaccessible to target proteases (PDB ID 20AY). Residue numbering was done according to mature C1-INH protein. Pictures were drawn by Dr. Christine Gaboriaud, Institut de Biologie Structurale, Grenoble France.

As a serpin (clade G), it regulates serine proteases *via* an irreversible suicide-substrate mechanism as a set mousetrap (25). C1-INH consists of 3 β-sheet structures, 9 α-helices, and a Reactive Center Loop (RCL) located at the top of the central β-sheet (Figure 3A). RCL serves as a bait region for specific proteases. The target protease recognizes and cleaves the P1-P1′ scissile bond in the RCL, with an insertion of the hinge and RCL into the central β-sheet A as an additional strand 4A. This drives the covalently bound protease to the base of the serpin molecule (26, 27). After this conformational change, the serpin adopts a thermodynamically stable conformation, with an irreversible inhibition of the protease. C1-INH-protease complexes are then cleared by the liver (28). This remarkable conformational change represents a common feature for serpins and illustrates a conformational pathology (25, 29). C1-INH also directly interacts with native C1 to prevent the autoactivation of C1 (30), thereby inhibiting its own consumption.

Laboratory identification of C1-INH-HAE is challenging with a primary interest for physicians, with the need (i) to recognize a C1-INH dysfunction and (ii) to decide on a molecular diagnosis, e.g., for patients with HAE without family history or with records of inconsistent biochemical measurements.

C1-INH Inhibitor circulates in the plasma (0.21–0.35 g/L), corresponding to a nearly 100% serpin function measurement. Two types of C1-INH function assays are available, the residual enzymatic C1s protease/KKS activity (chromogenic assay) or the C1-INH-protease complex formation using an ELISA. The chromogenic assay is more sensitive than the ELISA for detecting a low C1-INH function and is often preferred (31, 32).

The genetic basis of C1-INH-HAE means that family history represents a starting point for diagnosis. So, all family members must be strongly encouraged to be tested when a C1-INH-HAE has been diagnosed in a relative.

### C1-INH Expression Criteria: Biological Phenotype

Variants commonly characterized as HAE-2 from the only normal antigenic C1-INH criterion are found within the RCL (i.e., variants affecting the positions Ala^443^-Arg^444^ in the mature sequence; Figure 3A) and at the positions Gln^201^ and Lys^251^ (20). Many missense variations meet the Rosen's criterion of HAE-2 (18) and affect the functionality after protein misfolding. When an abnormal, nonfunctional protein is synthesized, circulating C1-INH presents with a remnant 105-kDa species after incubation with equimolar C1s protease on the anti-C1-INH immunoblot (20). This analytical strategy is helpful to identify the molecular features of any missense variant, with HAE-2 characteristics, including latent and oligomerized forms (33).

### Haploinsufficiency

As C1-INH-HAE is inherited as a dominant disorder in heterozygous cases, with one normal allele, antigenic C1-INH should theoretically be 50%. However, common observations recognized that C1-INH values are <35% of normal (i.e., from analytical threshold to 35%); attacks of angioedema are likely to occur when functional C1-INH levels are within this range (34).

Haploinsufficiency is common in C1-INH-HAE, where a dominant-negative variant product impairs the expression of normal allele. Haploinsufficiency may occur either through a *trans*-inhibition of normal protein expression (e.g., in an intracellular retention subsequent to intermolecular aggregates), or with decreased C1-INH production due to altered epigenetic control (Section Dominant-Negative Effect).

Decreased C1-INH function in plasma could be caused by high catabolism of remaining C1-INH was demonstrated in a 95-kDa species in patient plasma (20). This molecular distribution is in line with an increased proteolysis, rather than with a haploinsufficiency.

### Limits of Biological Testing

Transient decreases of C1-INH function are recurrently found in *F12*-c.983C>A;p.(Thr328Lys) female carriers, with angioedema attacks precipitated by estrogen intake or pregnancy. This decrease is consistent with C1-INH proteolysis by activated KKS (35).

Variant identification could be essential for the HAE diagnosis of patients presenting with symptoms, but also with the biological features of AAE-C1-INH or autoimmunity and with the lack of HAE family history, as reported by Veronez (36).

### Homozygous and Compound Heterozygous Probands Carrying *SERPING1* Variants

Hereditary angioedema due to C1 Inhibitor deficiency dominant trait, though with incomplete penetrance in heterozygous probands, early suggested that homozygosity for *SERPING1* variants may be embryonically lethal despite the fact that no signs of increased developmental lethality are present in HAE cohorts (37). In the last 15 years, several HAE pedigrees evidencing a recessive manner of inheritance have been documented and to date account for 10 true homozygous probands and 11 compound heterozygote alleles segregating in nine pedigrees. In general, *SERPING1* variants with recessive behavior represent a minor percentage of the total (5.8%) and are commonly classified by pathogenicity prediction tools such as “benign,” “likely benign,” or “variant of uncertain significance (VUS).” They are rare in the general population, usually presenting with minor allele frequencies (MAFs) below 0.0001. No *SERPING1* region has been recognized as specifically linked to a recessive feature, the variants being distributed all over the gene. No specific mutation categories are statistically associated with homozygosity, with two promoter, six missense, one splicing, and one indel variants (Supplementary Table S1).

The variants c.[-(163)C>T] and c.[-(161)A>G] are located in the promoter region of *SERPING1* and disrupt a putative CAAT box located at −62 bp from the origin of transcription (38–40). These two variants provide a hypothetical mechanism for their pathogenicity based on the defective transcription of *SERPING1*. The disruption of CAAT promoter sequences hampers the transcription of the affected alleles due to a defective binding of the RNA polymerase II and other nuclear-binding factors. However, in the case of *SERPING1*, there exists no direct evidence of the functionality of its CAAT sequence and therefore additional mechanisms may be postulated (41). The remaining recessive *SERPING1* variants reported affect the coding sequence of the gene and account for one variant in the 5′ untranslated region and six variants distributed through exons 4, 7, and 8. The variant c.-21T>C in the second nucleotide of exon 2 was reported as a probable cause of the disease in a homozygous proband with a HAE-1 phenotype (42). Due to its relatively high MAF (0.03), it has been reported as a polymorphic or disease-modifying allele (38, 43–45).

The seven remaining homozygous variants located in the coding sequence are associated with a HAE-1 phenotype in symptomatic homozygotes and with a HAE-2 (most commonly) or an asymptomatic presentation in heterozygous carriers. An illustrative example of this segregation of phenotypes is the c.1385T>G;p.(Ile462Ser) variant. In the original pedigree described by Blanch et al., HAE manifested exclusively in one of the two homozygous siblings as a HAE-1 phenotype while all their heterozygous relatives remained asymptomatic despite presenting with C4 consumption and a low C1-INH function (37). Interestingly, the homozygous probands from this family, as well as those with the c.[1198C>T];p.(Arg400Cys) (46) and c.[1379C>T];p.(Ser460Phe) (47) variants, presented with a very low or an undetectable antigenic C1q, thus reminding the phenotype of some patients with acquired angioedema with C1-INH deficiency. This acquired C1q deficiency is not an invariant trait in patients with C1-INH homozygous deficiency but pertains to the specific functional impairments of some recessive variants (47). Other variants have been found in homozygous carriers (Supplementary Table S1): c.[440T>A];p.(Val147Gln), c.[668A>C];p.(Gln223Pro), c.1202T>C];p.(Ile401Thr), and [c.646_647delAinsTCAGTGTCGTG], and the latter is characterized as a *de novo* variant (48).

Compound heterozygosity of *SERPING1* alleles with highly variable and incomplete penetrance is also a cause of C1-INH-HAE, with pedigrees presenting with uncommon symptomatic individuals (Supplementary Table S1); one or both allele(s) has (have) been characterized as VUS/benign variant(s).

### Digenic Variants

On the other hand, the combined presence of a *SERPING1* variant and a variant in another HAE susceptibility gene is now recognized as a cause of the disease. Examples of such situations are the combination of *SERPING1* variants with the *F12*-c.-4C>T polymorphic variant (rs1801020; MAF 0.472) (49, 50) and that of the c.513 + A>G *SERPING1* variant with the *F12*-c.1032C>A;p(Thr328Lys) pathogenic variant presenting HAE symptoms since 7 years of age with a severe phenotype (51).

A curious case shows an interesting pattern of the localization of swellings in a family composed by patients carrying (i) the variant c.1480C>T;p.(Arg494^*^) in *SERPING1* in heterozygosis, (ii) the variant c.988A>G;p.(Thr328Lys) in *PLG* in heterozygosis, or (iii) both (52). Individuals carrying only the *SERPING1* mutation or the combination of *SERPING1* and *PLG* mutations presented abdominal pain and edema of the extremities (hands and feet) more frequently when compared to the patients carrying only the *PLG* variant. Records of patients with PLG-HAE present with a higher proportion of attacks affecting the tongue and face, and less abdominal attacks (53), which was confirmed in this family case study (52). The presence of *SERPING1* and *PLG* pathogenic variants demonstrated a combination of symptoms but was not enough to prove an increase in the severity of the disease phenotype.

In an attempt to explain and correlate the variability in the manifestation of symptoms in C1-INH-HAE with other genes, Veronez et al. (54) evaluated 45 *SERPING1* mutation carriers and 15 healthy relatives from 26 families. The authors analyzed 15 genes entangled in the function of KKS and metabolism of associated enzymes and ligands/receptors using a next-generation sequencing (NGS) panel, and a total of 211 different variants were identified in the 15 genes analyzed. *BDKRB2* and *CPM* presented a large number of variants in untranslated regions, whereas *ACE, CPM*, and *NOS3* genes presented a higher number of variants directly affecting amino acid sequence. Despite the large amount of variants identified, no specific variant was significantly associated to any of the clinical symptoms affecting the patients (facial, abdominal, extremities, upper airways, and genitalia), indicating that the modulation of HAE symptoms could require a more complex regulation, probably involving pathways beyond the KKS, epigenetics, and environmental factors.

Aiming to uncover the genetic basis of nl-C1-INH-HAE, Loules et al. also applied a custom NGS platform to analyze 55 genes related to KKS involved in angioedema pathogenesis (55). Patients with normal antigenic C1-INH were evaluated in the study using patients with C1-INH-HAE as control, deciphering the presence of common variants that could modulate the patient clinical phenotype. Although the frequency of variants per gene was comparable between HAE with normal C1-INH function (nC1-INH-HAE) and C1-INH-HAE, variants of the *KNG1* and *XPNPEP1* genes were detected only in patients with nC1-INH-HAE. The authors concluded that alterations in some genes (e.g., *KNG1*) could play a role in the complex trait of HAE. These results emphasize the importance of modulator genes in HAE clinical expression with a better understanding of disease pathophysiology. This observation possibly drives the discovery of new therapeutic targets and provides useful indicators for disease clinical management.

### Polygenic/Oligogenic Conditions

Recently, the results suggest that polygenic situation is common in C1-INH-HAE and can significantly influence the penetrance of the disease, as exemplified by Veronez et al. (56). Almost all symptomatic patients from a pedigree presenting with C1-INH deficiency due to the c.1369G>C;p.(Ala457Pro) *SERPING1* variant carry multiple allele combinations with *ACE* [c.970C>T;p.(Arg324Trp)], *ENPEP* [c.638A>G;p.(Gln213Arg)], *KLK1* [c.433G>C;p.(Glu145Gln) or c.556A>G;p.(Lys186Glu)], *KLKB1* (c.428G>A;p.(Ser143Asn) or c.1679G>A;p.(Arg560Gln)], *KNG1* (c.533T>C;p.(Met178Thr) or c.591T>G;p.(Ile197Met)], *NOS3* (c.894T>G;p.(Glu298Asp) or c.2654G>T;p.(Arg885Met)] or *PRCP* (c.336A>T;p.(Glu112Asp)] alleles (56). This genetic complexity is pertaining with the present understanding of HAE as a pathology caused by an overactivation of the KKS and/or by a kinin accumulation.

## Distribution of Variants

The *ensembl* database displays 8,574 variants for the *SERPING1* gene (URL www.ensembl.org; ID ENSG00000149131; retrieved on October 28, 2021), with a homogeneous distribution along the 21,785 bases (GRCh38/hg38; latest assembly). Only 809 have been registered as pathogenic or likely pathogenic and 50 as VUS, according to the published observations and fulfilling the American College of Medical Genetics (ACMG) recommendations (57, 58). These variations are recorded in genetic databases (e.g., LOVD, tracked October 28, 2021; ClinVar, tracked November 2, 2021). Supplementary Table S2 shows the distribution of pathogenic/likely pathogenic variants.

### Missense Variants

Taking into account pathogenic/likely pathogenic/VUS variants, the distribution of missense variants is highly unbalanced between the mucin-like N-terminal domain (8/112 aminoacid residues; i.e., 7.1% of residues are affected) and the serpin domain (241/366; 65.8%); six variants have been recognized within the signal peptide sequence (6/22; 27.2%). This is congruent with a great impact of any modification of the peptide sequence within the serpin domain on C1-INH dysfunction. This prompts the data curator to a special attention for the interpretation of a missense variant with functional structures of strategic importance for serpin biology: gate, shutter, breach, hinge region, and less importantly, polyanion-binding domain (Figure 3A).

Missense variants (32.2% of all disease-causing variants) must be considered in association with mutant allele expression and C1-INH function. Most of the variant products are expressed, fulfilling criteria of a HAE-2 or an intermediate type, and have been distinguished between class I (i.e., altered exposure of the active site), class II (i.e., disturbed insertion of the RCL), and class III (i.e., conformational transition with spontaneous self or mutual insertion of the RCL) (20, 59) (Figures 2, 3); the latter could shape a M^*^ serpin conformation prior to the formation of extending chains of ordered and thermodynamically stable polymers, a characteristic of serpinopathy, or C1-INH species taking a stable and pathological latent form (Figure 3B) (25).

### Large Deletions/Insertions

Large deletions/duplications are likely to result from the recombination between *Alu* repeat sequences present in most introns of the *SERPING1* gene (60), and account for 8.3% of all disease-causing variants in the *SERPING1* gene (19; LOVD). The 7 introns contain 19 *Alu* repeat sequences, and a high density of *Alu* repeats has been reported in introns 3, 4, and 6. This is considered as a hotspot for nonhomologous recombination resulting in deletions or duplications, with exon 4 as the most affected (22, 60). Different approaches, including Southern blot analysis (61), fluorescent multiplex PCR (62–64), and long-range PCR (22, 65), have been used to detect those variants, while MLPA represents the reference technique for the detection of large deletions and insertions (22, 64, 65). The disadvantage of those methods is that it cannot precisely locate the boundaries of the insertion/deletion. To determine a precise location, time-demanding combination of previously mentioned methods with direct sequencing of the boundaries must be performed (64–66). Recently, a targeted NGS platform has been developed and validated to simultaneously detect the large deletions/insertions providing at the same time both exact size and location of the deletions/insertions (67). Up to now 64 large deletions/insertions in the *SERPING1* gene have been reported (19; LOVD), but for the majority, the exact size and location of boundaries remain unknown.

### Small Deletions/Insertions

Small deletions/duplications/insertions are abundant in *SERPING1* variants (36.2%) and mostly with subsequent frameshift and characterized as pathogenic/likely pathogenic.

### Variants Affecting Splicing

Splicing affecting variants typically have a deleterious effect on protein expression and/or function. They can result in exon skipping (or even multiple exon skipping), intron retention, *de novo* splice site creation, cryptic splice site usage, or a combination of 2 or more of these effects. All mentioned options were demonstrated to occur in *SERPING1* variants (68).

#### Variants at Intron/Exon Boundaries

Splicing mutations comprise 14.3% of *SERPING1* mutations, and 47% of them are located in conserved canonical positions (±1, ±2) of splice sites adjoining all exons of the gene (20) (Supplementary Table S5). According to HGMD and LOVD databases, 35 substitutions and 13 deletions and insertions changing canonical positions have been described as HAE-1-causing variants. Pathogenicity of these variants is well established and easily assessed by *in silico* prediction tools.

The evaluation of potentially pathogenic splicing variants in other than canonical positions is more difficult and considerably less reliable using *in silico* prediction tools; especially of those affecting splicing regulatory elements (SRE). Importantly, various procedures have been applied to prove their effect on splicing, from analyses of *SERPING1* variant transcript from patients' blood-derived RNA samples or from minigene splicing assays (62, 67).

Siddique et al. referred for the first time the correlation of an intronic variant with HAE in a family carrying c.10291 + G>T (69). The function of the variant could not be proven as the mRNA appeared to be normal. However, the wild-type mRNA had a relative abundance of ≈50%, suggesting that the mutant replicate was not converted to a stable mRNA and rapidly degraded.

**Figure 4:**
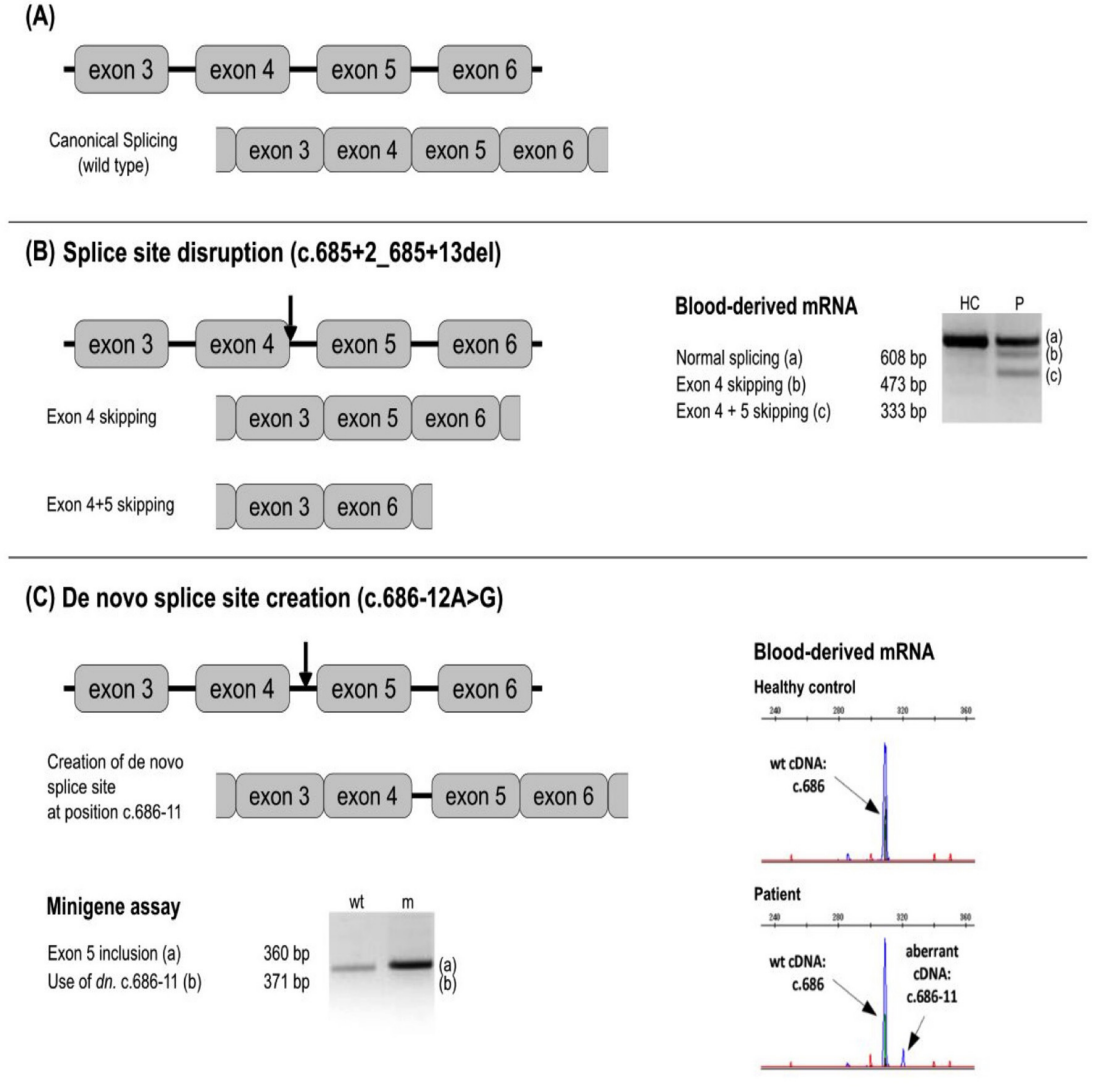
Examples of a splicing variant analysis. Next to the respective splicing defect schemes, the results of blood-derived patients' RNA analysis and/or minigen analysis are shown as RT-PCR amplicons visualized by agarose or capillary gel electrophoresis. Arrows depict approximate location of the analyzed variants. **(A)** No splicing defect. **(B)** Splice site disruption. The variant c.685 + 2_685 + 13del leads to the formation of new transcripts with exon 4, and exons 4 + 5 skipped. **(C)** New splice site creation. The variant at c.686-12 leads to the splice site gain at the position c.686-11 and the inclusion of intron 4 in the transcript. *In silico* prediction is helpful to identify splicing-affecting mutations prior to functional assays (71).

Up to now, 31 potentially pathogenic variants in noncanonical intronic positions of splice sites have been described. Whereas pathological impact of some of these variants has been well established based on functional tests or affirmative results of multiple prediction tools and/or the presence in several patients, other variants require further analysis of their functional significance. Illustrative examples are given by c.686-12A>G (44, 70), creating a *de novo* splice site in intron 4 (68) (Figure 4), c.52-10T>A in intron 2 after the observation of alternative short transcript species (72), and c.1250-13G>A in intron 7 after an *in silico* analysis (40).

Consequences of several new splicing variants already reported (20) could be thus evaluated as pathogenic/likely pathogenic, and the effect of others (e.g., c.890-8C>G) remains to be further established.

Interestingly, Colobran et al. detected c.6852 + T>A in intron 4. Functional studies of the mRNA demonstrated that this variant leads to the omission of exon 4 (73). Exon 4 consists of 135 bp (i.e., 45 codons); the lack of exon 4 corresponds to an in-frame deletion. Thus, the mutant allele could produce a protein lacking 45 amino acids. The levels of the mutant mRNA have been found to be very low compared to the wild-type, indicating that the mutant mRNA was degraded by one of the three surveillance pathways. In addition, the bioinformatic tool RNAfold® predicted a modification of the secondary structure. These data are consistent with the degradation of the mutant mRNA *via* the no-go degradation (NGD) pathway, which is associated with secondary structural features.

In ≈85% of cases, a G base is located in the fifth nucleotide of an intron (74). Variants at the +5 position are thought to significantly reduce the binding at the 5′ splice site to the complementary site at the U1snRNP particle, one of the first steps in the complex process of mRNA splicing (75). Consequently, the immediately preceding exon is omitted, followed by the activation of a deviating 5′ splice site and complete retention of introns (76). Alternatively, variants at this site may result in a reduced quantity of wild-type mRNA, or qualitative defects, by omitting an exon, activating a cryptic splice site, or creating a new splice site. The variant c.-22-19_-22-4del was detected in intron 1, predicted as pathogenic by the bioinformatic tool MutationTaster**®**, but marked as VUS, until a functional study and/or other evidence proves its pathogenic significance (77). At c.550-5 in intron 3, both variants c.551-5T>A and c.550T>G have been characterized as pathogenic due to its location near the 3' splice site (Supplementary Table S5). The c.890-14C>G variant has been detected in intron 5 (78) and the c.1250-13G>A variant in intron 7 (40, 79), the latter reducing the possibility of splicing at a rate similar to a variant located at the canonical positions. Other examples are displayed in Supplementary Table S5: c.515 + G>A, c.5505 + G>C, c.5505 + G>A, c.12495 + G>A, and c.12495 + G>T.

**Figure 5:**
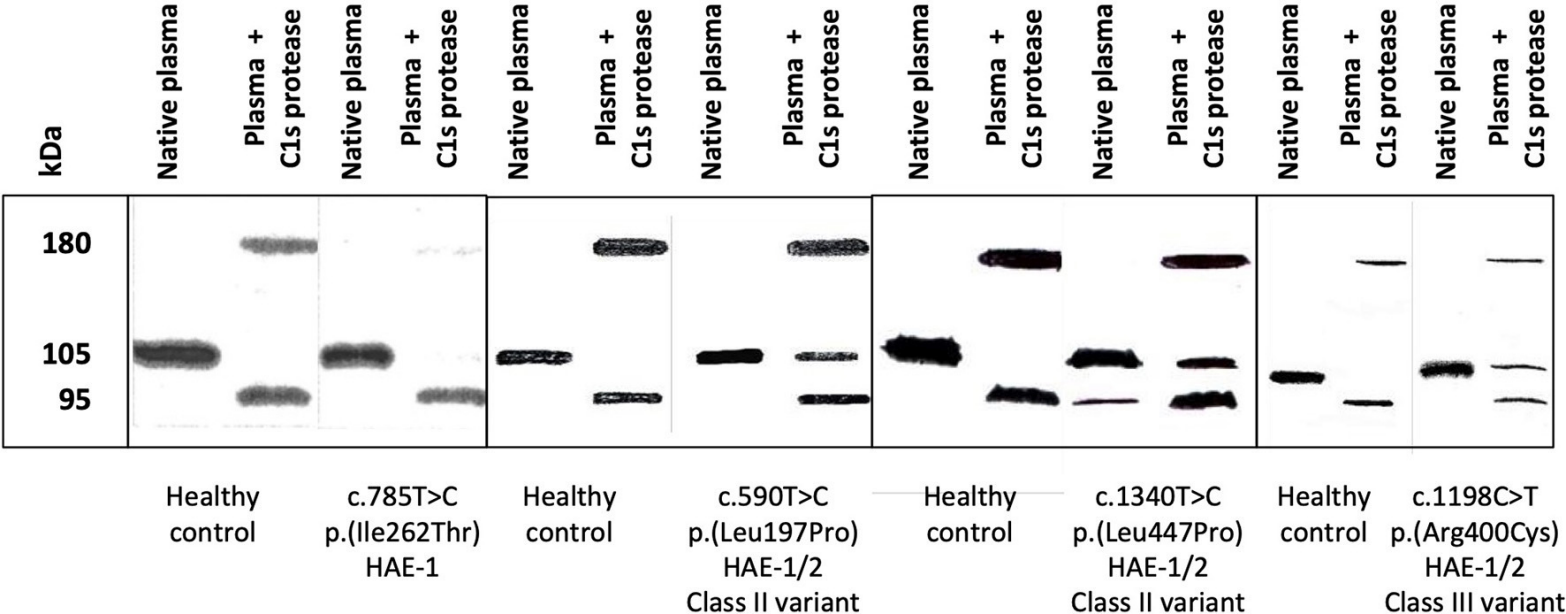
Circulating molecular species were displayed by an anti-C1-INH immunoblot assay. Citrate-plasma samples were collected from patients presenting with hereditary angioedema with C1 Inhibitor deficiency (C1-INH-HAE). Native plasma and plasma submitted to a 15-min incubation at 37°C with 0.1 nM C1s protease (plasma + C1s protease) were analyzed as described in (20). Four assays are displayed corresponding to healthy controls and patients with C1-INH-HAE: one type-I HAE (HAE-1) variant and three HAE-1/-2 variants, distributed in classes II and III. HAE-1/-2, C1-INH-HAE satisfying Rosen's criterion (18), with the expression of both alleles and presenting with a low antigenic C1-INH. C1-INH, C1 inhibitor.

Exonic variants can also affect splicing. The impact of several exonic variants on splicing has been functionally evaluated using the RNA analysis. Three substitutions in the last nucleotide of the exon 3 (position c.550), the deletion of the last nucleotide of the exon 4 (c.685del) (Figure 5), and substitutions in the proximity of the 3′ end of the exon 5 (c.882C>G, c.884T>G) have been shown pathogenic (63, 68). Moreover, the exonic variant c.-21T>C activates a cryptic acceptor site and causes exon 2 skipping to a certain extent (80) and might be linked to a more severe phenotype when occurring in *trans* with another pathogenic variant (20, 80).

#### Deep-Intronic Variants (>20 nt Upstream/Downstream of the Exon)

Near the 3' splice site, a pattern of polypyrimidines (C or T, 5–40 nucleotides) is usually located between the branch point and the 3′ splice site. As stated in Section Variants at Intron/Exon Boundaries, consequences of new variants within introns need additional consideration, in particular, the influence of the c.6853 + 1G>A variant (72).

Four variants have been detected deeper within the *SERPING1* gene and have been associated with HAE, two being considered as deep-intronic variants (i.e., located ≈100 bp from the intron–exon junction). The variant c.1029 + 84G>A in intron 6 is recurrent, detected in the affected families, and thought to affect the transcriptional process (44, 81–84). The bioinformatics analysis predicted that c.1029 + 384A>G results in a *neo-*5' splice site with the incorporation of a pseudo-exon into the transcript. The inclusion of the pseudo-exon leads to an early termination codon and possibly to the destruction of the produced mRNA by cell control mechanisms, which could potentially explain the reduced amount of proteins. The functional study performed by RT-PCR finally proved the above prediction (83). Vatsiou et al. detected the variant c.-22-155G>T in intron 1 in four patients (85). The *in silico* analysis predicted that the variant c.-22-155G>T causes a deleterious effect on the gene and degradation of the mutated transcript by the mRNA surveillance pathways; barring this variant was demonstrated by the loss of heterozygosity on the cDNA level. The variant was classified as pathogenic, in line with ACMG-AMP 2015 guidelines (e.g., Intervar) (57).

## Inheritance of Variants

### Dominant-Negative Effect

Dominant-negative involves a change of the function; the disease is not caused due to the loss of protein function, but happens due to a change in protein function or in disequilibrated mRNA species between alleles. The dominant-negative variant acts antagonistically in the wild-type allele by impairing its expression or by biochemically interacting with the normal gene product and interfering with C1-INH function.

A first pioneering investigation on C1-INH transcripts has shown a “*trans*” inhibition of the normal allele by variant mRNA or protein (86). The authors studied C1-INH expression in fibroblasts in which the mutant and wild-type mRNA and protein could be distinguished because of the deletion of exon 7. In patient cells, the wild-type mRNA was expressed at ≈50% of normal, whereas the mutant mRNA was 17% of normal. Rates of synthesis of both normal and mutant proteins were 11% and 3% of normal, respectively (i.e., lower than predicted from the mRNA levels), suggesting a *trans*-inhibition of normal allele by a variant product. A substantial reduction of both mRNA as well as C1-INH protein expression has also been shown using two allele-specific PCR in a carrier of the variant c.^*^101_^*^254del, a 155-bp deletion 100 bp downstream of the stop codon in exon 8 (87).

An elegant demonstration of dominant-negative effect of variants has been provided by Haslund et al. (88), where the mutated C1-INH species affected wild-type C1-INH in a dominant-negative manner with intracellular/plasmatic C1-INH aggregates and subsequent reduction in functional C1-INH. C1-INH encoded by a subset of HAE-causing *SERPING1* alleles disturbed the secretion of normal C1-INH protein in a dominant-negative fashion by triggering the formation of protein–protein interactions between normal and mutant C1-INH. The authors observed large intracellular C1-INH aggregates that were trapped in the endoplasmic reticulum. Interestingly, the transfection of wild-type *SERPING1* constructs into cells was able to remove the block in normal C1-INH secretion (88). Polymerogenic variants have been recognized with a location in proximity to/within the insertion site of RCL in C1-INH (i.e., shutter and hinge/gate regions; Supplementary Table S3), for example within α-helix C, c.566C>A;p.(Thr189Asn), β-sheet 3A, c.878T>C;p.(Ile293Thr) (33), α-helix F, c.838_846del;p.(Leu281_Ser283del) (88). The first variant prone to oligomerization has been recognized with c.1372G>A;p.(Ala458Thr) within RCL (89) and c.818_820del;p.(Lys273del) affecting the inter-domain α-helix F/β-sheet 3A (90). More recently, an additional variant p.(Ser150Phe) targeting α-helix A has been shown to be stably expressed within the cultured cells and not secreted into the medium at all. The mutant C1-INH significantly prevented the secretion of wild-type C1-INH, with its degradation within the cytoplasm through an interaction with the mutant protein (91). Observations of protease-resistant mutant C1-INH species suggest circulating latent C1-INH species that could also represent stable and low energetic conformations (Figure 3B, Supplementary Table S3) (20, 92).

### Recessive Variants

Some variants have been shown to circulate in a 50% expression of normal C1-INH allele, with an absence of haploinsufficiency (Supplementary Table S4). They do not segregate with symptomatic carriers. Homozygous carriers have been recognized as symptomatic for angioedema or these variants may have a disease-modifying effect (Section Homozygous and Compound Heterozygous Probands Carrying *SERPING1* Variants).

The recurrent c.1198C>T;p.(Arg400Cys) variant has been studied for its remittent expression (93). It is responsible for a temporary drop of C1-INH function with an enhanced effect in a homozygous state to express a HAE phenotype (recessive inheritance; 45). Once correctly folded, C1-INH^Arg378Cys^ is secreted as an active, although quite unstable, monomer. However, it could bear a folding defect, occasionally promoting protein oligomerization and interfering with the secretion process. Environmental factors (i.e., temperature, pH, and oxidative stress), which could apply even in situations of mild physical stress, like hyperthermia or metabolic acidosis, have been demonstrated *in vitro* to affect the stability and the function of C1-INH^Arg378Cys^.

Rare variants of *SERPING1* yielding deficient inhibitory activity toward complement C1 proteases but not toward the KKS proteases result in paucisymptomatic HAE. These have been characterized within the RCL [e.g., variations at the P2 position c.1394C>T;p.(Ala465Val) (94), at the P6 position c.1382C>T;p.(Ala461Val) (95, 96)], or out of the RCL [e.g., c.452T>G;p.(Leu151Arg) (20, 97)]. The position P2 in C1-INH supports protease specificity, with a significant reduction in rate constants for the reaction of C1-INH^Ala443Val^ with C1r, but not with C1s, FXIIa, plasma kallikrein or plasmin (96). However, C1-INH^Ala439Val^ (i.e., a variation at P6) displays a moderate decrease in control of C1s, compared with the wild-type protein (96).

### Uniparental Disomy

Gonadal mosaicism situations have rarely been detected in C1-INH-HAE; this condition must be distinguished from a *de novo* variant. The identification of mosaicism is important in establishing the disease diagnosis, assessing recurrence risk and genetic counseling. The pathogenic variant could be inherited from a maternal or paternal allele.

#### From a Maternal Allele

A c.[597C=/>G] condition has been found in a family in which only both sons, but not the parents, show clinical and laboratory findings typical of HAE, with allele segregation demonstrated using DHPLC (98). c.597C>G variant has not been detected in DNA derived from buccal cells, urinary cells, hair roots, and cultured fibroblasts from the mother, whereas it has occurred on the maternal transmitted chromosome.

#### From a Paternal Allele

1. Family 1. A c.[69_139=/del] has been shown in a family where three sons, but not the parents, show clinical and laboratory findings typical of HAE, with demonstrated allele segregation (99). c.69_139del variant has not been detected in DNA derived from somatic cells from the father and the mother, whereas it has occurred on the paternal transmitted chromosome.
2. Family 2. A c.[536C=/>T] has been observed in a family in which only both sisters, but not the parents, show clinical and laboratory findings typical of HAE, with allele segregation demonstrated using Sanger sequencing (100). c.536C>T variant has not been detected in DNA derived from lymphocytes from the father and the mother, whereas it is present on the DNA prepared from the sperm of the father and on the paternal transmitted chromosome.

## Noncausal Variants as Gene Modifiers

The combination of genetic variants may explain the variability in the manifestation of symptoms in C1-INH-HAE, in which nonpathogenic variants in diverse genes may confer susceptibility to a more severe phenotype when associated to pathogenic mutations in *SERPING1*. Within *SERPING1*, the c.-21T>C variant has been recognized as a disease modifier (Sections Homozygous and Compound Heterozygous Probands Carrying *SERPING1* Variants and Variants at Intron/Exon Boundaries). Beyond the known pathogenic mutations in *F12* gene that are described as causative for HAE (c.983C>A, c.983C>G, c.971_10182 + 4del, c.892_909dup), a common polymorphism, c.-4T>C (*F12*-46C/T) is demonstrated to influence the severity of disease in patients with C1-INH-HAE (50). This variant was associated with a delay in the onset of symptoms and with a decreased necessity to use long-term prophylaxis therapy (50, 101). A further study confirmed low FXII serum levels in patients with C1-INH-HAE carrying the T allele and found that asymptomatic patients presented the T allele in a higher frequency compared to symptomatic ones (102). Another study sequenced the exonic and regulatory regions (5′-UTR and 3′-UTR) of *F12* gene from 161 C1-INH-HAE and 191 HAE-nC1-INH, and found 6 *F12* polymorphisms in patients with C1-INH-HAE and 9 in patients with HAE-nC1-INH, including rare and first described variants (103). Variants such as *F12*-c.1768T>G;p.(Cys590Gly), which affect the catalytic domain of *F12* in a hotspot previously associated with protein deficiency, could be beneficial to HAE genetics. However, more studies are needed to establish any protective association of this variant in HAE (54).

## Data Curation

### *In silico* Rating

Although the genetic screening of the *SERPING1* gene has been facilitated by recent high-throughput technologies that have been available for massive DNA sequencing, clinical classification of the detected variants remains challenging. *SERPING1* variants could be divided into categories according to their possible pathogenicity:

- All variants (nonsense, frameshift, splicing, and large defects) with structural changes or misfolding of the protein, likely associated with a deleterious impact.
- Missense variants and changes affecting untranslated sequences in the 5′ or 3′ ends, both lacking strong evidence regarding their pathogenicity.

Pathogenicity supporting evidence provided by large pedigrees is required, such as functional analyses, population data, *in silico* predictions, and segregation family studies. Bioinformatic tools (e.g., SIFT®, PolyPhen®, Mutation Taster®) have been developed based on evolutionary conservation, the type of amino acid change, and the position within a functional domain, allele frequency. The certainty with which any detected variant is considered clinically relevant falls within a spectrum, ranging from pathogenic to unrelated to the phenotype.

Germenis et al. introduced a specific customization of ACMG criteria for *SERPING1* to improve variant interpretation of *SERPING1* variants (58). Every detected variant should be assessed with respect to its presence in public, internal and disease-specific databases, population data, computational predictions, *in vivo* and *in vitro* test results, evidence of segregation, and allelic and variant-specific information. The ACMG criteria that are supporting or tolerable must be applied to the abovementioned evidence, whenever possible, resulting in variant classification in one category (i.e., benign, likely benign, VUS, likely pathogenic, and pathogenic) (57). Variants and supporting data should be submitted to public databases upon classification, new evidence that may alter the initial variant assessment and favoring further exchanges between submitters. Online bioinformatic tools can be helpful for determining pathogenicity (e.g., Genetic Variant Interpretation Tool, InterVar, Varsome).

About variants detected in introns, many potential SRE-affecting variants fall into the category of so-called “variants of unknown significance” (104). To distinguish between pathogenic/likely pathogenic mutations and harmless non-splicing-affecting variants, medical geneticists are encouraged to investigate *in vitro* on transcript distribution from patient RNA samples or, although less reliable, to use *in silico* predictions (105). An estimate of exon susceptibility to be skipped or to activate nearby cryptic splice sites can be possible by SRE predictions. An evaluation of their reliability and potential use in clinical diagnostic settings has been developed by Grodecká et al. (71).

### Functional Analysis of Serpin Function

Establishing a dysfunctional C1-INH protein is a prerequisite for further genetic analysis. The common C1-INH function testing should be advantageously completed by an analysis of circulating species for missense variants.

When variants are recognized in an intronic region out of a noncanonical splice sequence, a transcription analysis of an RNA sample extracted from blood nucleated cells is recommended for a functional distinction between a benign and a pathogenic/likely pathogenic variant. Cultured monocytes provide RNA samples of good quality for a downstream analysis; however, the distribution of transcripts could be different from that extracted from blood nucleated cells. A specific approach taking into account a possible NMD of transcripts with premature stop codon should be used to minimize a risk of missing aberrant transcript when evaluating RNA extracted from patients' blood cells (83).

### Structural Analyses With Genotype to Phenotype Correlation; Conserved Positions Among Serpins

C1 Inhibitor controls target proteases as a suicide-substrate where RCL displays the scissile bond Arg^444^-Thr^445^ (i.e., P1-P1′; Figure 3A). The N-terminal region of RCL is conserved among inhibitory serpins, maintaining proper RCL mobility and loop insertion. However, the sequences adjacent to the cleavage site in the RCL, P6-P4′ (i.e., Ala^439^-Val^448^), are highly variable between the different serpins and considered to be a major determinant of serpin specificity (106). In this region, some variants have been recognized as benign/likely benign (e.g., those at the positions Ala^439^, Val^442^, or Val^448^); variants at nonadjacent positions have been characterized as pathogenic because a mutant on the hinge/RCL sequence packs favorably in the loop-inserted latent structure (e.g., those at the positions Ala^436^ or Pro^454^ located in the proximal and distal hinge, respectively; Figure 3B). Importantly because of a lack of the suicide-substrate property of C1-INH, all variants at position Arg^444^ are interpreted as pathogenic, inconsistently with commonly used *in silico* predictions.

If the protruding structure of the RCL makes it more accessible for an interaction with target proteases, it also favors the native serpin to be in a stressed thermodynamically metastable conformation M^*^. However, polymerized C1-INH represents a stable and low energetic conformation, which can be achieved upon some missense mutations (107). Variants have been recognized as favoring the thermodynamically stable conformation, with susceptibility to spontaneous loop-sheet polymerization or latent serpin species, as described in Section Inheritance of Variants (Supplementary Table S3). These variants, whose feature makes it recognized in the class III identified in Section Missense Variants, should be included as HAE-2. Figure 5 shows an example with the p.(Arg400Cys) variant.

### Frequency of Variants Within Global or Selected Population

Pertaining to the characterization of pathogenic criteria with C1-INH-HAE incidence of 0.00002, submitters are invited, whenever possible, to consider MAF (i.e., MAF in a global/selected population) (57).

**Figure 6:**
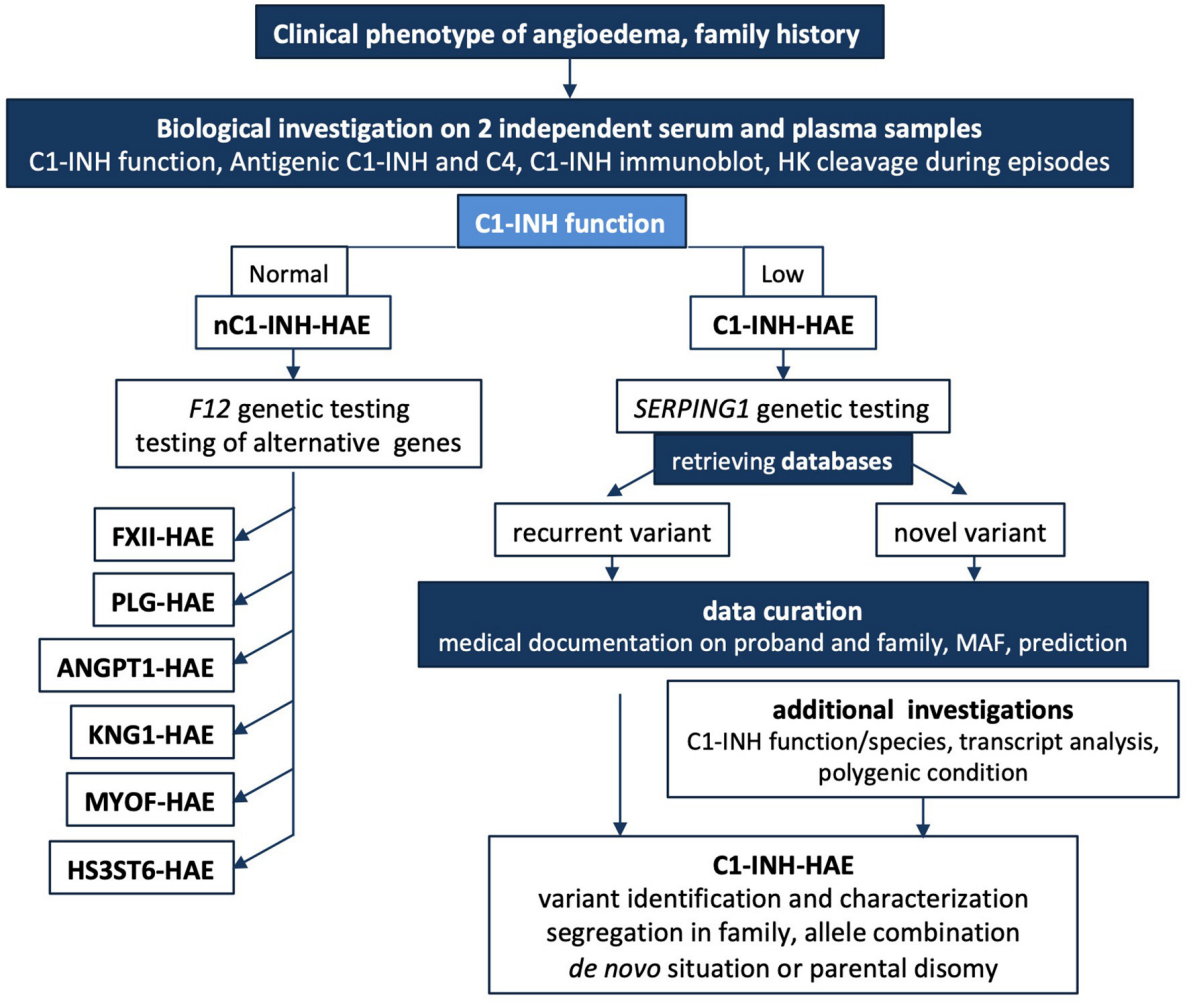
Short algorithm for C1-INH-HAE. The medical algorithm of Caballero et al. (108) is supplemented for biological and genetic additional testings. In cases where there is a family history of HAE or the clinical history is highly suggestive of HAE, biological investigation should begin with an assessment of the established C1-INH function after 2 independent determinations on plasma; this mandatory testing can be completed by C1-INH molecular species and in some instances by high molecular weight kininogen cleavage to establish the involvement of KKS. A low antigenic C4 in serum could contribute to biological diagnostic, but false positives should be considered because *C4A* and *C4B* null alleles are common. A normal C1-INH function could suggest a nC1-INH-HAE and *F12* genetic study should be performed. When unsuccessful, alternative genetic testing could be developed. *SERPING1* genetic testing can confirm a C1INH-HAE diagnosis. Medical geneticists investigate a possible *de novo* mutation or a parental disomy on additional DNA analyses. In case of intronic variant detection, transcript distribution is investigated for noncanonical sequences. Data curation is mandatory for every novel variant submitted to sequence interpretation according to ACMG guidelines (57, 58). C1-INH-HAE, hereditary angioedema with C1-INH deficiency; nC1-INH-HAE, hereditary angioedema with a normal C1-INH function; F12-HAE, with gain-of-function of FXII; PLG-HAE, with gain-of-function of plasminogen; ANGPT1-HAE, with altered angiopoietin-1; KNG1-HAE, with high molecular weight kininogen susceptible to cleavage; HS3ST6-HAE, with altered heparan sulfate-glucosamine 3-*O*-sulfotransferase 6; MYOF-HAE, with altered myoferlin; HK, high molecular weight kininogen; MAF, minor allele frequency.

Figure 6 shows a short algorithm displaying the position of biological and genetic analyses within a diagnostic algorithm.

### Contribution to the Field Statement

To our knowledge, this is the largest study taking an overlook of the constellation of *SERPING1* variants found in nearly 1,500 HAE families. This study emphasizes that etiopathogenesis of C1-INH-HAE could be consistently implemented by C1-INH molecular analyses.

Misfolding and polymerization/latentization of the mutated serpins are at the base of a group of conformational diseases collectively known as serpinopathies (25, 29, 107). Likewise, missense mutations in *SERPING1* can cause polymerization/latentization, with an impaired secretion or a failed cleavage by target protease, that lead to C1-INH deficiency and C1-INH-HAE (20, 33, 88, 89).

Recording *SERPING1* genetics together with biological data on serpin function and C1-INH transcripts, for intronic variants out of canonical sequences, should contribute to a high quality value for national registries and for open databases.

## Conclusion and Perspective

Hereditary angioedema due to C1 Inhibitor deficiency has been first characterized as a monogenic disease; however, cumulative arguments on a contribution of additional alleles make clear that clinical variability of C1-INH-HAE is substantially attributed to modifier genes. As suggested by Veronez et al. (36), even if pharmacogenomic associations are very difficult to prove in a rare disease, recommendations will assist the physician for an optimal treatment option for individual patients.

A pathogenic model of C1-INH-HAE has been proposed with a KKS activation in a systemic activation process, where fluid-phase activation of the KKS generates BK, associated with local manifestations after an interaction with locally expressed endothelial kinin receptors (11). The model provides an explanation for why symptoms can occur at multiple sites during an attack and why HAE attacks respond well to modest increases of circulating C1-INH function. The recent observations of multiple allele combinations and of abnormal kinin metabolism are congruent with this model, making C1-INH a strategic component with the participation of additional parameters in patient clinical phenotype.

Functional studies of modulating factors, acting on systemic activation or on activator-bound process, should combine with the discovery of new mutations, with genotype to biological phenotype associations and including a large number of patients.

The document presented here describes the biological and structural features of C1-INH deficiency in relation to groups of *SERPING1* variants. As well as inherited serpinopathies, namely, α1-trypsin deficiency, could be considered as ideal candidates for gene therapy, strategies for C1-INH-HAE treatment have been suggested. Based on the argument that HAE should be viewed primarily as a metabolic liver disorder, new therapeutic approaches to C1-INH-HAE have been outlined by Ameratunga et al. (109). Given the very high costs of treating HAE, the authors have considered that gene therapy as curative option may become feasible in the next decade.

In a next future, epigenetics and environmental factors should be considered in the molecular identification of C1-INH-HAE with the characterization of individual severity risk factors as well.

## Databases and Bioinformatics Supports

- ClinVar: www.ncbi.nlm.nih.gov/clinvar/
- Ensembl: http://www.ensembl.org, a centralized resource for geneticists
- Exonic splicing enhancers: ESEfinder release 3.0
- Genetic Variant Interpretation Tool: http://www.medschool.umaryland.edu/Genetic_Variant_Interpretation_Tool1.html
- Human Splicing Finder, version 3.1: www.umd.be>hsf
- InterVar: https://wintervar.wglab.org/evds.php
- LOVD: Leiden Open Variation Database; databases.lovd.nl/shared/variants/SERPING1
- Varsome: https://varsome.com/,InterVar,~wintervar.wglab.org.

## Author Contributions

CD, AL-L, AGh, ML-T, SC, DP, MR, TF, CV, JP, and AGe wrote sections or subsections of this manuscript. DP and AGh prepared Figure 5. TF and HG prepared Figure 4. FP prepared Supplementary Tables S2, S3. HG prepared Supplementary Table S5. CD, AL-L, AGh, ML-T, SC, DP, MR, TF, CV, JP, and AGe revised the last version of this manuscript. CD supervised the writing of the manuscript and the selection of the figures. All authors contributed to the article and approved the submitted version.

## Funding

TF and HG were supported by grants NV18-05-00330 (Ministry of Health) and MUNI/A/1099/2019 (Ministry of Education, Youth and Sports, Czech Republic).

## Conflict of Interest

AGh was employed by KininX SAS. FP and AGe were employed by CeMIA SA. The remaining authors declare that the research was conducted in the absence of any commercial or financial relationships that could be construed as a potential conflict of interest.

## Publisher's Note

All claims expressed in this article are solely those of the authors and do not necessarily represent those of their affiliated organizations, or those of the publisher, the editors and the reviewers. Any product that may be evaluated in this article, or claim that may be made by its manufacturer, is not guaranteed or endorsed by the publisher.

## Abbreviations

ACMG: American College of Medical Genetics
BK: bradykinin
C1-INH: C1 Inhibitor
C1-INH-HAE: Hereditary angioedema due to C1 Inhibitor deficiency
HAE: Hereditary angioedema
HAE-1: Hereditary angioedema type I
HAE-2: Hereditary angioedema type 2
KKS: Kallikrein–kinin system
MAF: Minor Allele Frequency
nC1-INH-HAE: HAE with normal C1-INH function
RCL: Reactive center loop
SRE: Splicing regulatory element
VUS: variant of uncertain significance.
