# Supplementary material for: SERPING1 Variants and C1-INH Biological Function: A Close Relationship With C1-INH-HAE
Source: Front Allergy. 2022 Mar 31;3:835503. doi: 10.3389/falgy.2022.835503 (PMC9361472; doi:10.3389/falgy.2022.835503)
Supplement: Supplementary file 1 [file Table_1.DOCX]

Table S1. Variants of *SERPING1* gene carried by homozygous and compound heterozygous probands. Amino acid numbering is according to immature protein sequence. NA: MAF value not affected

| **cDNA numbering^1^** | **Protein** | **Serpin structure^2^** | **Residue conservation^3^** | **dbSNP** | **Minor allele frequency^4^** | **Clinical/Biological significance^5^** | **Reference** |
| --- | --- | --- | --- | --- | --- | --- | --- |
| c.[-(163)C>T];[-(163)C>T] |  |  | - | *rs1387768389* | 3.18^E-05^ | Putatively disrupts CAAT box, with reduced C1-INH transcription.  One homozygous proband with HAE-I, asymptomatic heterozygous carriers | (37) |
| c.[-(161)A>G];[-(161)A>G] |  |  | - | *rs766344850* | 3.19^E-05^ | Putatively disrupts CAAT box.  One homozygous proband with HAE-I, asymptomatic heterozygous individuals. | (38) |
| c.[-21T>C];[-21T>C] |  |  | - | *rs28362944* | 0.0291 (single allele) | Splicing defect due to exon 2skipping. Confers severity when combined in *trans* with another *SERPING1* variant.  One homozygous proband with HAE-1, asymptomatic relatives. | (41) |
| c.[440T>A];[440T>A] | p.(Val147Glu) | α-helix A (shutter) | - |  | NA | One homozygous proband with HAE-1, asymptomatic heterozygous parents | (111) |
| c.[646_647delAinsTCAGTGTCGTG];[646_647delAinsTCAGTGTCGTG] | p.(Lys216Serfs*4) |  | - |  | NA | One homozygous proband with a *de novo* variant and HAE-1 | (47) |
| c.[668A>C];[668A>C] | p.(Gln223Pro) | α-helix E/ β-sheet 3A | - |  | NA | One homozygous proband with HAE-2, paucisymptomatic/asymptomatic heterozygous relatives | (19) |
| c.[1198C>T];[1198C>T] | p.(Arg400Cys) | β-sheet 2C/ β-sheet 6A (gate) | 72% | *rs201363394* | 1.39^E-05^ | Recurrent variant. One homozygous proband with HAE-1, paucisymptomatic/asymptomatic heterozygous individuals, with remittent HAE-2 | (45,92) |
| c.[1202T>C];[1202T>C] | p.(Ile401Thr) | β-sheet 6A  (gate) | - | *rs1263371770* | 0.4^E-05^ | Two homozygous probands with HAE-1, Heterozygous relatives with HAE-1, HAE-2 or asymptomatic phenotype | (112) |
| c.[1379C>T];[1379C>T] | p.(Ser460Phe) | RCL^6^  Variant at P8 | - |  | NA | Two homozygous probands with HAE-1, heterozygous relatives with HAE-1 | (112) |
| c.[1385T>G];[1385T>G] | p.(Ile462Ser) | RCL^6^  Variant at P5 | - | *rs763451792* | 2.0^E-05^ | One homozygous proband with HAE-1 phenotype, asymptomatic heterozygous relatives | (35) |
| c.[(-100)C>G;816_818del] | p.(Asn272del) |  | - |  | NA | Proband with HAE-1; c.-100C>G affects a pyrimidine-rich region (c.-108 to -77) of potential H-DNA structure; recurrent pathogenic c.816_818del variant | (37) |
| c.[5C>T(;)1045C>T] | p.(Ala2Val)(;)  (Leu349Phe) | Signal peptide; β-sheet 2B/ β-sheet 3B (breach) | -/76% (Leu^327^) | *rs1853462631*/*rs141075266* | 125.1^E-05^/6.4^E-05^ | Proband with HAE-1 ; association between two VUS ; incomplete penetrance of c.5C>T variant ; asymptomatic heterozygous c.1045C>T carriers | (19) |
| c.[48T>G;51+3A>G] | p.(Ala16=);(0?) |  | - |  | NA | Proband with a HAE-1 ; c.51+3A>G *cis-*linked to the synonymous polymorphism c.48T>G | (64) |
| c.[203C>T];[800C>T] | p.(Thr68Ile);(Ala267Val) |  | - |  | NA | Compound heterozygotes present with the highest severity score ; symptomatic c.203C>T carriers ; paucisymptomatic c.800C>T carriers | (113) |
| c.[820A>G(;)856C>T] | p.(Ile274Val);p.(Arg286Cys) |  | - |  | NA | Symptomatic proband with a HAE-2; symptomatic heterozygous carriers of c.820A>G, paucisymptomatic c.856C>T carriers | (19) |
| c.[856C>T(;)1361T>A] | p.(Arg286Cys);p.(Val454Glu) |  | - |  | NA | Symptomatic proband with a HAE-2; recurrent c.1361T>A variant; Val^432^ critical for serpin function (hinge), symptomatic carriers of c.1361T>A | (114) |
| c.[889+3A>G(;)1249+4A>G] |  |  | - |  | NA | Compound heterozygote with a low mRNA expression ; asymptomatic single heterozygous carriers | (115) |
| c.[922A>G(;)1030-20A>G] | p.(Thr308Ala) |  | -/84% (Thr^286^) | *rs1803212(;)rs2511988* | NA | Symptomatic proband; p.(Thr308Ala) found polymorphic by MutationTaster®; c.1030-20A>G characterised as benign ;  asymptomatic single heterozygous carriers | (84,116) |
| c.[1029+84G>A(;)1396C>G] | p.(Arg466Gly) | RCL^6^  Variant at P1 | - |  | NA | Highly symptomatic proband presenting with a HAE-2; symptomatic carriers of c.1029+84G>A | (81) |
| c.[1282T>C;1342G>C] | p.(Cys428Arg);p.(Glu448Gln) |  | - |  | NA | Symptomatic proband with a HAE-1; Cys to Arg transition destroys the disulfide bridge with Cys101 suggesting NAan unstable serpin ; C1-INH^Glu226Gln^ prone to oligomerize | (79) |
| c.[1420C>G;1442T>G] | p.(Gln474Glu);p.(Leu481Arg) |  | - |  | NA | p.(Gln452Glu) has little/no effect on C1-INH structure or function (benign variant), whereas *in vitro* secretion of C1-INH^Leu459Pro^ is abolished. | (37) |

^1^ Coding sequence numbering is according to cDNA sequence of *SERPING1* (Ensembl Gene ENSG00000149131; NCBI RefSeq NM_000062.2), where c.1 is the A of the ATG initiating codon and c.1503 is the A of the TGA stop codon.

^2^ Identification of structural characteristics within the C1-INH overall structure as displayed on the 3D model of C1-INH (PDB ID 5DU3; Figure 3)

^3^ Residue conservation among serpins; aminoacid residues strictly conserved in >70% serpin sequences (n=219)

^4^ Minor Allele Frequency (MAF) according to Genome Aggregation Database (gnomAD)

^5^ Records by authors or from NCBI ClinVar ressources (www.ncbi.nlm.nih.gov/clinvar/).

^6^ Reactive Site Loop of serpins (RCL), essential for protease recognition and RCL mobility and conformational transformation for its insertion as neo-strand 4A (Figure 3).

**REFERENCES**

111. Guryanova I, Suffritti C, Parolin D, Zanichelli A, Ishchanka N, Polyakova E, et al. Hereditary angioedema due to C1 inhibitor deficiency in Belarus: epidemiology, access to diagnosis and seven novel mutations in SERPING1 gene. *Clin Mol Allergy*. (2021) 19:3. doi: 10.1186/s12948-021-00141-0

112. Kawachi Y, Hibi T, Yamazaki S, Otsuka F, A. novel donor splice site mutation in the C1 inhibitor gene of a patient with type I hereditary angioneurotic edema. *J Invest Dermatol*. (1998) 110:837–9. doi: 10.1046/j.1523-1747.1998.00170.x

113. Suffritti C, Zanichelli A,Maggioni L, Bonanni E, CugnoM, CicardiM. Highmolecular-weight kininogen cleavage correlates with disease states in the bradykinin-mediated angioedema due to hereditary C1-inhibitor deficiency. *Clin Exp Allergy*. (2014) 44:1503–14. doi: 10.1111/cea.12293

114. Jaradat SA, Caccia S, Rawashdeh R,MelhemM, Al-Hawamdeh A, Carzaniga T, et al. Hereditary angioedema in a Jordanian family with a novel missense mutation in the C1-inhibitor N-terminal domain. *Mol Immunol*. (2016) 71:123–30. doi: 10.1016/j.molimm.2016.02.001

115. Xu Y-Y, Zhi Y-X, Yin J, Wang L-L, Wen L-P, Gu J-Q, et al. Mutational spectrum and geno-phenotype correlation in Chinese families with Hereditary Angioedema. *Allergy*. (2012) 67:1430–6. doi: 10.1111/all.12024

116. Faiyaz-Ul-Haque M, Al-Gazlan S, Abalkhail HA, Al-Abdulatif A, Toulimat M, Peltekova I, et al. Novel and recurrent mutations in the C1NH gene of Arab patients affected with hereditary angioedema. *Int Arch Allergy Immunol*. (2010) 151:149–54. doi: 10.1159/000236005
