# Supplementary material for: SERPING1 Variants and C1-INH Biological Function: A Close Relationship With C1-INH-HAE
Source: Front Allergy. 2022 Mar 31;3:835503. doi: 10.3389/falgy.2022.835503 (PMC9361472; doi:10.3389/falgy.2022.835503)
Supplement: Supplementary file 2 [file Table_2.DOCX]

Table S2. Distribution of pathogenic/likely pathogenic variants in *SERPING1* (LOVD database).

| **Variant type** | **n** | **%** |
| --- | --- | --- |
| missense | 259 | 32.1 |
| nonsense | 72 | 8.9 |
| large rearrangements | 66 | 8.2 |
| small deletion/insertion | 293 | 36.2 |
| affecting splicing | 116 | 14.3 |
| 3’ sequence | 1 | 0.1 |
| 5’ sequences | 2 | 0.2 |
| **total** | **809** |  |
