# Supplementary material for: SERPING1 Variants and C1-INH Biological Function: A Close Relationship With C1-INH-HAE
Source: Front Allergy. 2022 Mar 31;3:835503. doi: 10.3389/falgy.2022.835503 (PMC9361472; doi:10.3389/falgy.2022.835503)
Supplement: Supplementary file 3 [file Table_3.DOCX]

Table S3. Dominant-negative variants. The variants, where molecular mechanism is a direct effect of a proneness to misfold due to the metastability of the native conformation, have been characterized in the class III (19,88). Amino acid numbering is according to immature protein. NA, dbSNP ID not attributed.

| **cDNA numbering^1^** | **Protein** | **Serpin structure^2^** | **Residue conservation^3^** | **Minor allele frequency^4^** | **dbSNP** | **Clinical/Biological significance^5^** | | **Reference**  **ClinVar** |
| --- | --- | --- | --- | --- | --- | --- | --- | --- |
| c.449C>T | p.(Ser150Phe) | α-helix A (shutter) | - |  | NA | prone to oligomerisation |  | (41,90,110,117) |
| c.508T>C | p.(Ser170Pro) | β-sheet 6B (shutter) | 93% |  | NA | prone to partially polymerise with normal C1-INH |  | (87) |
| c.530T>C | p.(Leu177Pro) | α-helix B (shutter) | - |  | NA | prone to polymerise with normal C1-INH |  | (87) |
| c.550G>A | p.(Gly184Arg) | α-helix B/ α-helix C | 80% | <1^E-05^ | *rs281875170* | Recurrent variant  prone to polymerise with normal C1-INH | Circulating oligomers | (32,117) |
| c.(550+1_551-1)_(685+1_686-1)del  (exon 4 del) | p.(Gly184_Pro228del) | loss of α-helix D, α-helix C and β-sheet 2A, opening the shutter domain | - |  | NA | Recurrent deletion variant  prone to polymerise with normal C1-INH |  | (87) |
| c.566C>A | p.(Thr189Asn) | α-helix C (shutter) | 87% |  | NA | prone to polymerise with normal C1-INH | Circulating oligomers | (32,87,117) |
| c.707T>C | p.(Phe236Ser) | α-helix E | 75% |  | NA | prone to polymerise with normal C1-INH |  | (87) |
| c.773A>T | p.(Asn258Ile) | α-helix E (shutter) | - |  | NA | prone to polymerise with normal C1-INH | Circulating oligomers | (87) |
| c.816_818del | p.(Asn272del) | β-sheet 3A (shutter) | 89% |  | NA | Variant affecting one of the three glycosylation sites of the serpin domain  prone to oligomerisation/latentisation |  | (91) |
| c.818_820del | p.(Lys273del) | α-helix F/ β-sheet 3A (shutter) | - |  | NA | Acquisition of a *neo* N-glycosylation site. | Circulating oligomers | (89) |
| c.838_846del | p.(Leu281_Ser283del) | β-sheet 3A (shutter) | - |  | NA | prone to polymerise with normal C1-INH | Circulating oligomers | (87) |
| c.863_864delinsAA | p.(Val288Glu) | β-sheet 3A (shutter) | - |  | NA | prone to polymerise with normal C1-INH |  | (87) |
| c.871A>C | p.(Asn291His) | β-sheet 3A (shutter) | 85% |  | *rs1057520366* | prone to polymerise with normal C1-INH |  | (87)  ClinVar ID VCV000378573.3 |
| c.878T>C | p.(Ile293Thr) | β-sheet 3A (shutter) | - |  | *rs1590826571* |  | Circulating oligomers | (32) |
| c.(1029+1_1030-1)_(1249+1_1250-1)del  (exon 7 del) |  |  | - |  | NA | *trans-*inhibition of normal C1-INH allele |  | (85)  ClinVar ID VCV000003944.2 |
| c.1372G>A | p.(Ala458Thr) | RCL^6^ | - |  | *rs121907947* | Recurrent variant  aligned to P10 of A1AT  prone to oligomerisation/latentisation  C1-INH^Ala436Thr^ not cleavable by target proteases |  | (19)  ClinVar ID VCV000003945.2 |
| c.1322T>C | p.(Met441Thr) | β-sheet 5A (shutter) | - |  | *rs281875175* | prone to oligomerisation/latentisation |  | (87)  ClinVar ID VCV000068245.1 |
| c.1342G>C | p.(Glu448Gln) | β-sheet 1C (gate) | - |  | NA | prone to oligomerisation |  | (87) |
| c.1427C>T | p.(Pro476Leu) | β-sheet 1C/β-sheet 4B (gate) | 96% |  | NA | Recurrent variant  prone to oligomerisation |  | (87) |
| c.1466C>G | p.(Pro489Arg) | β-sheet 5A (shutter) | - |  | NA | prone to oligomerisation/latentisation |  | (91) |
| c.1478G>A | p.(Gly493Glu) | β-sheet 5A (shutter)  strategic position  for shutter function | 89% |  | NA | prone to oligomerisation/latentisation |  | (91,117) |
| c.1493C>T | p.(Pro498Leu) | Distal hinge region (gate) | 95% |  | NA | Recurrent variant  prone to oligomerisation/latentisation |  | (91,117) |

^1^ Coding sequence numbering is according to cDNA sequence of *SERPING1* (Ensembl Gene ENSG00000149131; NCBI RefSeq NM_000062.2), where c.1 is the A of the ATG initiating codon and c.1503 is the A of the TGA stop codon.

^2^ Identification of structural characteristics within the C1-INH overall structure as displayed on the 3D model of C1-INH (PDB ID 5DU3)

^3^ Residue conservation among serpins; aminoacid residues strictly conserved in >70% serpin sequences (n=219)

^4^ Minor Allele Frequency (MAF) according to Genome Aggregation Database (gnomAD)

^5^ Records from authors or from NCBI ClinVar ressources (www.ncbi.nlm.nih.gov/clinvar/).

^6^ Reactive Site Loop of serpins (RCL), essential for protease recognition and RCL mobility and conformational transformation for its insertion as neo-strand 4A (Figure 3)

**REFERENCES**

110. Hashimura C, Kiyohara C, Fukushi J-I, Hirose T, Ohsawa I, Tahira T, et al. Clinical and genetic features of hereditary angioedema with and without C1-inhibitor (C1-INH) deficiency in Japan. *Allergy*. (2021) 76:3529–34. doi: 10.1111/all.15034

117. Mete Gökmen N, Rodríguez-Alcalde C, Gülbahar O, Lopez-Trascasa M, Onay H, López-Lera A. Novel homozygous variants in the SERPING1 gene in two Turkish families with hereditary angioedema of recessive inheritance. *Immunol Cell Biol*. (2020) 98:693–9. doi: 10.1111/imcb.12362
