# Supplementary material for: SERPING1 Variants and C1-INH Biological Function: A Close Relationship With C1-INH-HAE
Source: Front Allergy. 2022 Mar 31;3:835503. doi: 10.3389/falgy.2022.835503 (PMC9361472; doi:10.3389/falgy.2022.835503)
Supplement: Supplementary file 4 [file Table_4.DOCX]

Table S4. Some examples of variants of the *SERPING1* gene characterised as recessive and benign. Amino acid numbering is according to immature protein sequence. ESE, exonic splicing enhancer; ESS, exonic splicing silencer; NA, dbSNP ID not attributed; VUS, variant of uncertain significance.

|  | **cDNA numbering^1^** | **Protein** | **Serpin structure^2^** | **Residue conservation^3^** | **Minor allele frequency^4^** | **dbSNP** | **Clinical/Biological significance^5^** | **Reference**  **Clin Var** |
| --- | --- | --- | --- | --- | --- | --- | --- | --- |
| Recessive variants | c.452T>G | p.(Leu151Arg) | α-helix A (shutter) | - | - | NA | VUS (heterozygous) | (19) |
|  | c.1382C>T | p.(Ala461Val) | RCL^6^  Variant at P6 | - | - | NA | Pathogenic when combined with c.820A>G or c.1361T>A | (95) |
|  | c.1394C>T | p.(Ala465Val) | RCL^6^  Variant at P2 | - | - | *rs121907950* | Likely benign (heterozygous) | (95) |
| Introducing/disrupting intronic or exonic splicing regulatory elements | c.52-130C>T |  |  |  | 0.388 | *rs1005510* | Pathogenic when combined with c.5C>T | (64) |
|  | c.129G>T | p.(Gly43=) |  |  | 25.1^E-05^ | *rs149573972* | The G to T transition creates an ESS and disrupts an ESE | (70) |
|  | c.468C>T | p.(Ala156=) |  |  | 101.0^E-05^ | *rs150601964* | Segregates with symptomatic patients in a family carrying multi-allelic combinations | (55) |
| Pathogenic from *in silico* bioinformatics applications | c.461A>G | p.(Tyr154Cys) | α-helix A (shutter) |  | - | *rs281875168* | Nearly normal control of KKS | (90) |
|  | c.509C>T | p.(Ser170Phe) | β-sheet 6B (shutter) | 93% | - | *rs281875169* |  | (110,114) |
|  | c.695T>A | p.(Ile232Lys) | β-sheet 2A/ α-helix E | - | - | *rs281875172* |  | (117,118) |
|  | c.1289T>A | p.(Leu430Gln) | α-helix I/ β-sheet 5A | - | - | *rs281875174* |  | (118) |
|  | c.1475T>C | p.(Met492Thr) | β-sheet 5B (shutter) | - | 0.4^E-05^ | *rs978962357* |  | ClinVar ID VCV000427759 |
| Activation of a cryptic acceptor site with exon 2 skipping | c.-21T>C |  |  |  | 0.02908 | *rs28362944* | Asymptomatic heterozygous carriers of c.-21T>C variant.  c.-21T>C might be considered as a gene modifier, conferring severity when combined in *trans* with another *SERPING1* pathogenic variant | (75) |

^1^ Coding sequence numbering is according to cDNA sequence of *SERPING1* (Ensembl Gene ENSG00000149131; NCBI RefSeq NM_000062.2), where c.1 is the A of the ATG initiating codon and c.1503 is the A of the TGA stop codon.

^2^ Identification of structural characteristics within the C1-INH overall structure as displayed on the 3D model of C1-INH (PDB ID 5DU3; Figure 3)

^3^ Residue conservation among serpins; aminoacid residues strictly conserved in >70% serpin sequences (n=219)

^4^ Minor Allele Frequency (MAF) according to Genome Aggregation Database (gnomAD)

^5^ Records by authors or from NCBI ClinVar ressources (www.ncbi.nlm.nih.gov/clinvar/).

^6^ Reactive Site Loop of serpins (RCL), essential for protease recognition and RCL mobility and conformational transformation for its insertion as neo-strand 4A

**REFERENCE**

118. Sekijima Y, Hashimoto T, Kawachi Y, Koshihara H, Fujio Otsuka F, Ikeda S-I, et al. novel RNA splice site mutation in the C1 Inhibitor gene of a patient with type I hereditary angioedema*. Intern Med*. (2004) 43:253–5. doi: 10.2169/internalmedicine.43.253
