# Supplementary material for: SERPING1 Variants and C1-INH Biological Function: A Close Relationship With C1-INH-HAE
Source: Front Allergy. 2022 Mar 31;3:835503. doi: 10.3389/falgy.2022.835503 (PMC9361472; doi:10.3389/falgy.2022.835503)
Supplement: Supplementary file 5 [file Table_5.DOCX]

Table S5. Pathogenic and likely pathogenic variants of the *SERPING1* gene identified within introns (up to 20 nucleotides upstream and downstream from the 5’ and 3’ end of the exon, respectively) and at intron-exon boundaries, in a selection for their participation in the transcript splicing. *In silico* prediction tools for *SERPING1* analysis of splicing regulatory elements are described in (105). All carriers of the variants have been found symptomatic. NA, dbSNP ID not attributed; NMD, nonsense-mediated decay.

| **Position** | **cDNA numbering^1^** | **Minor allele frequency^2^** | **dbSNP** | **Clinical/Biological significance^5^** | **Evaluation** | **Reference** |
| --- | --- | --- | --- | --- | --- | --- |
| Exon 1 | c.-22-2A>C |  | NA | Affecting the canonical acceptor site and possibly a correct transcriptional process. | pathogenic | (119) |
|  | c.-22-2A>G |  | NA |  | pathogenic | (44) |
|  | c.-22-2A>T |  | NA |  | pathogenic | (19) |
|  | c.-22-1G>A |  | NA |  | pathogenic | (41) |
| Intron 2 | c.49_51+13del |  | NA | Deletion overlapping the canonical donor site. | pathogenic | (19) |
|  | c.50_51+6del |  | NA | Affecting the canonical donor site, with demonstrated complete exon 2 and partial exon 3 skipping. | pathogenic | (64) |
|  | c.51+1G>A | 0.6^E-05^ | *rs1470120365* | Affecting the canonical donor site. | pathogenic | (43,77) |
|  | c.51+1G>T |  | NA |  | pathogenic | (53,117) |
|  | c.51+2T>A |  | NA |  | pathogenic | (19) |
|  | c.51+2T>C |  | NA |  | pathogenic | (53,110,111) |
|  | c.51+3A>G |  | NA | Noncanonical substitution. Affecting intron 2 in a noncanonical position; demonstrated exon 2 and 3 skipping. | pathogenic | (19,43,62,78,120) |
|  | c.51+3A>C |  | NA | Noncanonical substitution. Likely affecting the donor site, with exon 2 skipping. | pathogenic | (19,50) |
|  | c.51+3A>T |  | NA |  | pathogenic | (112) |
|  | c.51+5G>A |  | *rs1554994665* | Noncanonical substitution. Likely affecting intron 2 in a non-canonical position; exon 2 partially skipped. | pathogenic | (37,78) |
|  | c.51+5G>T |  | NA | Noncanonical substitution. Likely affecting intron 2 in a non-canonical position. | pathogenic | (121) |
|  | c.51+6T>G |  | NA | Noncanonical substitution. Low transcription of the full-length isoform.  c.51+6T>G variant probably causes a deviated transcription pathway, with degradation of the full-length transcript. | pathogenic | (64) |
|  | c.52-10T>A |  | NA | Noncanonical substitution. c.-52-10T>A causes an alternative short transcript species. | pathogenic | (68) |
|  | c.52-2del |  | NA | Deletion affecting the canonical acceptor site. | pathogenic | (60) |
|  | c.52-2A>G |  | NA | Affecting the canonical acceptor site, with exon 3 skipping. | pathogenic | (43) |
|  | c.52-1G>A | 1.4^E-05^ | *rs886041353* | Affecting the canonical acceptor site, with exon 3 skipping. | pathogenic | (77,117) |
| Exon 3 | c.550G>A |  | *rs281875170* | Substitutions in the last nucleotide of the exon 3 affecting the function of acceptor splice site with exon 3 skipping. | pathogenic | (19,37,44,53,60,62,67,117,122) |
|  | c.550G>C |  | NA |  | pathogenic | (19,39,44,53,62) |
|  | c.550G>T |  | NA |  | pathogenic | (67) |
| Intron 3 | c.550+1G>A |  | NA | Affecting the canonical donor site. | pathogenic | (19,123) |
|  | c.550+2T>C |  | *rs112666115* |  | pathogenic | (19,62,76) |
|  | c.550+2dup |  | NA |  | pathogenic | (124) |
|  | c.550+5G>A |  | NA | Noncanonical substitution. Loss of relatively conserved G nt (80% conservation) at donor splice site, affecting the canonical donor site. | pathogenic | (62) |
|  | c.550+5G>C |  | NA |  | pathogenic | (62) |
|  | c.551-5T>A |  | NA | Noncanonical substitution. Affecting the canonical acceptor site. | pathogenic | (77) |
|  | c.551-3C>G |  | NA | Noncanonical substitution. Affecting the canonical acceptor site. | pathogenic | (77) |
|  | c.551-2A>C |  | *rs113574262* | Affecting the canonical acceptor site. | pathogenic | (120) |
|  | c.551-2A>G |  | NA | Affecting the canonical acceptor site, with demonstrated exon 4 skipping. | pathogenic | (19,67,77) |
|  | c.551-2del |  | NA | Deletion affecting the canonical acceptor site. | pathogenic | (19,62) |
|  | c.551-1dup |  | NA | Insertion affecting the canonical acceptor site. | pathogenic | (66) |
|  | c.551-1G>A |  | NA | Affecting the canonical acceptor site. | pathogenic | (76,120) |
|  | c.551-1G>C |  | NA |  | pathogenic | (117,119) |
|  | c.551-8_558del |  | NA | Deletion overlapping the canonical acceptor site. | pathogenic | (19) |
| Intron 4 | c.685+1del, duplicated |  |  | Deletion affecting the canonical donor site, with demonstrated skipping of exon 1 and exons 4 and 5 | pathogenic | (67) |
|  | c.685+1G>A |  | *rs113263597* | Affecting the canonical donor site. | pathogenic | (19,77,91,118) |
|  | c.685+1G>T |  | NA | Affecting the canonical donor site. | pathogenic | (43,70) |
|  | c.685+1delG |  | NA | Deletion of the last nucleotide of exon 4 affecting the function of the donor site, with demonstrated skipping of exon 4 and exons 4 and 5. | pathogenic | (67) |
|  | c.685+2T>A |  | NA | Affecting the canonical donor site, with demonstrated partial exon 4 skipping and in-frame deletion p.(Gly217_Pro228del). | pathogenic | (71) |
|  | c.685+2T>G |  | NA | Affecting the canonical donor site. | pathogenic | (43) |
|  | c.685+2_685+13del |  | NA | Deletion affecting the canonical donor site. | pathogenic | (67) |
|  | c.685+5G>A |  | NA | Noncanonical substitution. Affecting the canonical donor site. | pathogenic | (19) |
|  | c.685+5G>T |  | NA |  | pathogenic | (19) |
|  | c.686-12A>G |  |  | Noncanonical substitution. Creating a *de novo* splice site in intron 4, with demonstration of an aberrant transcript and subsequent p.(Asp229Alafs*8) and NMD. | pathogenic | (43,67,69) |
|  | c.686-3C>G |  | NA | Noncanonical substitution. Loss of the canonical acceptor site, with demonstrated exon 5 skipping. | pathogenic | (62) |
|  | c.686-1G>A |  | NA | Affecting the canonical acceptor site. | pathogenic | (35,117) |
| Exon 5 | c.882C>G |  | NA | Substitutions in the proximity of the 3′ end of the exon 5, probably causing defect in ESE, demonstrated as affecting the function of acceptor site with a skipped exon 5. | pathogenic | (19,62) |
|  | c.884T>G |  | NA |  | pathogenic | (62) |
| Intron 5 | c.889+1del |  | NA | Deletion affecting the canonical donor site. | pathogenic | (19) |
|  | c.889+1G>A |  | NA | Affecting the canonical donor site. | pathogenic | (19,117) |
|  | c.889+1G>T |  | NA | Affecting the canonical donor site. | pathogenic | (77) |
|  | c.889+2T>C |  | NA | Affecting the canonical donor site, with demonstrated exon 5 skipping, with an in-frame deletion and expected protein expression p.(Asp229_Ser296del). | pathogenic | (62,91) |
|  | c.889+3A>T |  | NA |  | pathogenic | (122) |
|  | c.889+4_+8del |  | NA | Deletion of noncanonical sequence. Affecting the canonical donor site. | pathogenic | (66, 76) |
|  | c.890-14C>G |  | NA | Affecting the canonical acceptor site. | pathogenic | (66, 76) |
|  | c.890-2A>G |  | NA | Affecting the canonical acceptor site. | pathogenic | (120) |
|  | c.890-1G>A |  | NA |  | pathogenic | (124) |
|  | c.1029+1G>A |  | NA | Affecting the canonical donor site. | pathogenic | (19,78,77) |
|  | c.1029+1G>T |  | NA | Affecting the canonical donor site, with lower wild-type mRNA to ≈50%, suggesting a mutant not converted to a stable mRNA and rapidly degraded. | pathogenic | (68) |
|  | c.1029+2T>G |  | NA | Affecting the canonical donor site. | pathogenic | (19) |
|  | c.1029+3_1029+6del |  | NA | Deletion of noncanonical sequence. Affecting the canonical donor site. | pathogenic | (77) |
|  | c.1030-2A>G |  | NA | Affecting the canonical acceptor site. | pathogenic | (117) |
|  | c.1029+4del |  | NA | Hypothetically affecting intron 6 in a noncanonical site. | likely pathogenic | (44, 53) |
|  | c.1030-12_1046del |  | NA | Deletion comprising polypyrimidine tract. | pathogenic | (37) |
|  | c.1030-10_1048del |  | NA | Deletion comprising polypyrimidine tract. | pathogenic | (19) |
|  | c.1030-2_1031delinsGCA |  | NA | Deletion comprising polypyrimidine tract. | pathogenic | (37, 49) |
|  | c.1030-1G>C |  | NA | Affecting the canonical acceptor site, with demonstrated exon 7 skipping. | pathogenic | (37,64,77,117) |
|  | c.1030-1G>A |  | NA | Affecting the canonical acceptor site. | pathogenic | (49, 66, 116, 117) |
|  | c.1030-1G>T |  | NA | Affecting the canonical acceptor site. | pathogenic | (19) |
|  | c.1030-1del |  | NA | Deletion of noncanonical sequence. Affecting the canonical acceptor site. | pathogenic | (118) |
| Intron 7 | c.1249+1G>A |  | NA | Affecting the canonical donor site, with demonstrated exon 7 skipping. | pathogenic | (35,64,77) |
|  | c.1249+1G>T |  | *rs112565881* | Affecting the canonical donor site. | pathogenic | (19) |
|  | c.1249+1G>C |  | NA |  | pathogenic | (64) |
|  | c.1249+2del |  | NA | Deletion affecting the canonical donor site, with demonstrated exon 7 skipping, 50% reduction of the mRNA, no mutant mRNA detected, indicating a highly unstable mutant mRNA. | pathogenic | (62) |
|  | c.1249+2T>A |  | NA | Affecting the canonical donor site. | pathogenic | (125) |
|  | c.1249+4A>G |  |  | Hypothetically affecting intron 7 in a noncanonical site. | likely pathogenic | (19) |
|  | c.1249+5G>A |  | NA | Noncanonical substitution. Demonstrated to affect the canonical donor site, with exon 7 skipping. | pathogenic | (19,67,71,75) |
|  | c.1249+5G>T |  | NA |  | pathogenic | (43) |
|  | c.1250-13G>A |  | NA | Noncanonical substitution. An *in silico* analysis gives the c.1250-13G>A variant as likely to create a *neo*-acceptor splice site. | pathogenic | (39,77) |
|  | c.1250-2A>G |  | NA | Affecting the canonical acceptor site. | pathogenic | (77) |
|  | c.1250-1G>A |  | NA |  | pathogenic | (119) |

^1^ Coding sequence numbering is according to cDNA sequence of *SERPING1* (Ensembl Gene ENSG00000149131; NCBI RefSeq NM_000062.2), where c.1 is the A of the ATG initiating codon and c.1503 is the A of the TGA stop codon. Existence of 2 transcripts (NM_000062.2 and NM_001032295.2) should be considered when evaluating variants in 5' UTR region of the *SERPING1* gene.

^2^ Minor Allele Frequency (MAF) according to Genome Aggregation Database (gnomAD)

^3^ Records by authors or from NCBI ClinVar ressources ([www.ncbi.nlm.nih.gov/clinvar/](http://www.ncbi.nlm.nih.gov/clinvar/)).

**REFERENCES**

119. Kalmár L, Bors A, Farkas H,Vas S, Fandl B,Varga L. et al.,Mutation screening of the C1 inhibitor gene among Hungarian patients with hereditary angioedema. *Hum Mutat*. (2003) 22:498–498. doi: 10.1002/humu.9202

120. Cumming S, Halsall DJ, Ewan PW, Lomas DA. The effect of sequence variations within the coding region of the C1 inhibitor gene on disease expression and protein function in families with hereditary angio-oedema. *J Med Genet*. (2003) 40:e114. doi: 10.1136/jmg.40.10.e114

121. Kang HR, Yim EY, Oh SY, Chang YS, Kim YK, Cho SH, et al. Normal C1 inhibitor mRNA expression level in type I hereditary angioedema patients: newly found C1 inhibitor gene mutations. *Allergy*. (2006) 61:260–4. doi: 10.1111/j.1398-9995.2006.01010.x

122. Johnsrud I, Kulseth MA, Rødningen OK, Landrø L, Helsing P, Nielsen EW, et al. A nationwide study of Norwegian patients with hereditary angioedema with C1 Inhibitor deficiency identified six novel mutations in SERPING1. *PLoS ONE*. (2015) 10:e0136011. doi: 10.1371/journal.pone.0136011

123. Brix ATH, Svensson TM, Sandberg M, Bygum A. Hereditary angioedema: the challenges of cross-border family investigation and treatment. *BMJ Case Rep*. (2020) 13:e231906. doi: 10.1136/bcr-2019-231906

124. Nabilou S, Pak F, Alizadeh Z, Reza Fazlollahi M, HoushmandM, AyaziM, et al. Genetic study of hereditary angioedema type I and type II (first report from Iranian patients: describing three new mutations). *Immunol Invest*. (2020) 1–12. doi: 10.1080/08820139.2020.1817068

125. Aabom A, Andersen KE, Fagerberg C, Fisker N, Jakobsen MA, Bygum A. Clinical characteristics and real-life diagnostic approaches in all Danish children with hereditary angioedema. *Orphanet J Rare Dis*. (2017) 12:55. doi: 10.1186/s13023-017-0604-6
